# Deciphering the genetic basis for vitamin E accumulation in leaves and grains of different barley accessions

**DOI:** 10.1038/s41598-019-45572-7

**Published:** 2019-07-01

**Authors:** Christian Schuy, Jennifer Groth, Alexandra Ammon, Julia Eydam, Steffen Baier, Günther Schweizer, Anja Hanemann, Markus Herz, Lars M. Voll, Uwe Sonnewald

**Affiliations:** 1 Division of Biochemistry, Department of Biology, Friedrich-Alexander-Universität Erlangen-Nürnberg, Staudtstr. 5, D-91058 Erlangen, Germany; 2 Institut für Pflanzenbau und Pflanzenzüchtung, Bavarian State Research Center for Agriculture, Am Gereuth 8, D-85354 Freising, Germany; 3 Saatzucht Josef Breun GmbH & Co. KG, Amselweg 1, D-91074 Herzogenaurach, Germany; 4 Division of Plant Physiology, Department Biology, Philipps-University Marburg, Karl-von-Frisch-Str. 8, D-35043 Marburg, Germany

**Keywords:** Metabolomics, Metabolic engineering, Agricultural genetics, Field trials, Molecular engineering in plants

## Abstract

Tocopherols and tocotrienols, commonly referred to as vitamin E, are essential compounds in food and feed. Due to their lipophilic nature they protect biomembranes by preventing the propagation of lipid-peroxidation especially during oxidative stress. Since their synthesis is restricted to photosynthetic organisms, plant-derived products are the major source of natural vitamin E. In the present study the genetic basis for high vitamin E accumulation in leaves and grains of different barley (*Hordeum vulgare* L.) accessions was uncovered. A genome wide association study (GWAS) allowed the identification of two genes located on chromosome 7H, *homogentisate phytyltransferase* (*HPT-7H*) and *homogentisate geranylgeranyltransferase* (*HGGT*) that code for key enzymes controlling the accumulation of tocopherols in leaves and tocotrienols in grains, respectively. Transcript profiling showed a correlation between *HPT-7H* expression and vitamin E content in leaves. Allele sequencing allowed to decipher the allelic variation of *HPT-7H* and *HGGT* genes corresponding to high and low vitamin E contents in the respective tissues. Using the obtained sequence information molecular markers have been developed which can be used to assist smart breeding of high vitamin E barley varieties. This will facilitate the selection of genotypes more tolerant to oxidative stress and producing high-quality grains.

## Introduction

Nearly 100 years ago the report of the existence of a hitherto unrecognized dietary factor essential for reproduction of mice led to the discovery of vitamin E^1^. The classification vitamin E nowadays refers to a group of eight structurally related lipophilic compounds. Four are classified as tocopherols and four as tocotrienols collectively known as tocochromanols^2^. The term tocopherol is an allusion regarding its necessity in the pregnancy of mice (Greek *tókos*: birth; and *phérein*: bearing) and was first proposed after the successful isolation of an alcohol (ending: -ol) from wheat germ oil with the properties of vitamin E^3^.

The structure of this alcohol was solved when the reaction product (later: α-tocopherol) of phytyl-bromide and trimethyl-hydroquinone displayed identical chemical properties as vitamin E and gave excellent activity in biological tests^4^. Common features of all tocochromanols are a hydrophobic polyprenyl side chain and a chromanol ring derived from homogentisate (HGA). The hydrophobic polyprenyl side chain anchors these compounds in biological membranes while a hydroxyl group at the chromanol ring confers vitamin E antioxidant activity: It can donate its phenolic hydrogen atom to scavenge radicals mainly to prevent propagation of lipid peroxidation in biological membranes (e.g. reviewed in^5^). While tocopherols comprise a saturated prenyl side chain the trivial name tocotrienol was chosen for tocochromanols with a tri-unsaturated aliphatic tail with double bonds at the 3′, 7′ and 11′ position^6^. Tocochromanols are exclusively synthesized by photosynthetic microorganisms like cyanobacteria and algae as well as plants^7^. While it is generally assumed that tocopherols are ubiquitously present in the plant kingdom tocotrienols are only present in certain plant groups^8^.

The first committed step in tocopherol biosynthesis the prenylation of HGA with the polyprenyl compound phytyl pyrophosphate (P-PP) is catalysed by a HGA phytyltransferase (HPT - VTE2) and was shown to take place in the chloroplast envelope membrane^9^. The first committed step in tocotrienol biosynthesis depends on an enzyme designated as HGA geranylgeranyltransferase (HGGT). It displays enhanced affinity towards the tri-unsaturated polyprenyl compound geranylgeranyl pyrophosphate (GG-PP) over the saturated P-PP^10^. The enzymatic reactions downstream of HGA prenylation were also shown to take place at the inner chloroplast envelope membrane^11^. Furthermore, they do not discriminate between compounds with saturated or unsaturated side chains as proven by the introduction of the barley HGGT enzyme into *Arabidopsis thaliana* which is usually free of tocotrienols^10^. The HPT product MPBQ (2-methyl-6-phytyl-1,4-benzoquinol) as well as the HGGT product MGGBQ (2-methyl-6-geranylgeranyl-1,4-benzoquinol) can serve as the substrate for a MPBQ methyltransferase (MPBQ-MT - VTE3) which creates 2,3-dimethyl-6-phytyl-1,4-benzoquinol (DMPBQ) and 2,3-dimethyl-6-geranylgeranyl-1,4-benzoquinol (DMGGBQ), respectively. All four benzoquinones can act as substrates for tocopherol cyclase (TC - VTE1) which adds a second oxygen-containing ring at the junction between the aromatic head group and the prenyl tail to create the two-ring structure known as a chromanol ring^12^. TC activity generates δ-tocopherol (δ-tocotrienol) from MPBQ (MGGBQ) and γ-tocopherol (γ-tocotrienol) from DMPBQ (DMGGPQ). These isoforms can eventually be methylated at the aromatic ring by a γ-tocopherol methyltransferase (γTMT - VTE4) to generate β-tocopherol (β-tocotrienol) or α-tocopherol (α-tocotrienol), respectively. The methylation status of the chromanol ring therefore distinguishes the four tocopherol and tocotrienol species^13^.

While the benzoquinol intermediates do not accumulate in notable amounts^14^ the substrate flow towards β- and α-tocopherol is not saturated *in planta* so that the progenitor isoforms δ- and γ-tocopherol can be observed in green parts^15^ and in plant-derived oils^16^ in a species- and tissue-specific manner. The ratio of the four tocopherol isoforms is influenced by the enzymatic activities and is especially well studied in *Arabidopsis thaliana* mutant and overexpression lines (reviewed in^17^). For the biosynthesis of tocochromanols the building blocks have to be provided by independent pathways. The aromatic compound HGA is derived from L-tyrosine. To this end the conversion from L-tyrosine to 4-hydroxyphenylpyruvate (HPP) is catalysed by a tyrosine aminotransferase (TAT) and from HPP to HGA by a HPP dioxygenase (HPD) which can reportedly limit tocopherol biosynthesis^18^. P-PP or GG-PP are possible products of the non-mevalonate pathway (DOXP pathway) and are in direct relation to each other via geranylgeranyl diphosphate reductase (GGDR) activity^19^. Desoxy-D-xylulose-5-phosphat-synthase (DXP) catalyses the first step of the DOXP pathway and a recent report speculates about a quantitative influence of DXP on the tocochromanol pool in maize^20^. Besides, it was long speculated that P-PP and GG-PP can derive from chlorophyll degradation *in vivo* which releases phytyl^21^. This could be proven when a phytyl kinase (PK - VTE5)^22^ and a phytyl phosphate kinase (PPK - VTE6)^23^ were identified and tested to have relevant influence on tocochromanol biosynthesis.

Historically, the genetic dissection of tocochromanol biosynthesis started with the identification of the *TC*^14^ (*vte1*) and *HPT*^24^ (*vte2*) in *Arabidopsis thaliana*. Since then, overexpression of structural pathway genes, whether endogenous or heterologous, was used to successfully and significantly increase the vitamin E content of many plant species and tissues (e.g. reviewed in^25^). To influence the vitamin E content by classical breeding one relies on the existence of natural genetic diversity in the germplasm of a species and the ability to validate the heredity of the desired trait. An elegant way to dissect this variability is the identification of quantitative trait loci (QTLs) by genome wide association studies (GWAS). QTL mapping uses statistical techniques to localize chromosomal regions that most likely contain genes contributing to phenotypic variation in a complex trait of interest and thus make it accessible for marker-assisted breeding efforts^26^. QTLs associated with tocochromanol content have been detected for several crop species including tomato^27^, rapeseed^28–30^, maize^20,31–33^, soybean^34,35^, rice^36,37^ and barley^38,39^.

Recently, we reported on a large-scale metabolic profiling approach with leaves from drought and heat stressed barley varieties grown under controlled conditions in phytochambers^39^. Overall 57 metabolites have been analysed including those of primary carbon and nitrogen as well as antioxidant metabolism. Parallel genotyping of the investigated accessions allowed the identification of QTLs for tocopherol and other metabolites for plants grown under various environmental conditions. In the present study we set out to scrutinize whether the previously identified chromosomal regions determine the vitamin E composition and accumulation under field conditions. To achieve this goal two new but partially overlapping sets of genetically diverse spring barley genotypes were compiled and consecutively grown and probed under simulated drought conditions in a rain out shelter (RS) in the field. The study allowed us to confirm the previously determined QTLs for vitamin E accumulation in barley leaves^39^ and grains^38^ and enabled us to identify the underlying structural genes. Comparing genomic sequences of the different genotypes resulted in the determination of allelic variation which could further be associated with different expression levels of one structural gene of vitamin E biosynthesis, *HPT-7H*, correlating to high or low vitamin E accumulation. The uncovered allelic variation can be used to select for high quality barley genotypes in the future.

## Results

### Validation of a tocopherol-QTL on chromosome 7H

A QTL located on the long arm of the barley chromosome 7H at 118–122 cM (centimorgan) was identified to be associated with γ-tocopherol content of flag leaves of barley plants grown in different controlled environmental conditions including single drought and combined drought and heat stress^39^. The first goal of the present study was to assess the stability of this association in field-grown plants. Therefore, two partially overlapping sets of spring barley genotypes were selected and analysed under drought and well-watered/irrigated conditions in the field. The new sets consisted of elite lines from North America and Germany as well as latest German breeding material and Mediterranean land races (please see Supplementary Table S3 for cultivars). In two consecutive years those new sets were grown successively in a mobile rain-out shelter (RS) to simulate drought stress under field conditions as described in the methods section. In brief, well-watered control conditions were achieved by controlled artificial irrigation in two of the four plots in the RS while the rest was not watered at all. Soil moisture measurements with tensiometers in different depths verified that the water availability was below the wilting point (−500 hPa) during heading stage (BBCH-stage 59–77) in the drought subjected plots (Supplementary Fig. S1).

Leaf samples of flag leaves minus one were taken at heading stage and tocochromanol contents were analysed by high performance liquid chromatography (HPLC). To assess genetic diversity of the investigated barley varieties at the chromosomal region of interest we utilized the KASP-method (Competitive Allele Specific PCR) to specifically determine the single nucleotide polymorphism (SNP) patterns of three SNP-markers originally found in the QTL^39^. In agreement with our previous work we confirmed significantly elevated contents of γ-tocopherol and additionally of α-tocopherol in genotypes carrying one of the two detected SNP haplotypes. This correlation was stable across growing seasons (2016 and 2017), genotype sets (2016 and 2017) and growth conditions (well-watered and drought) (Table 1) which supported our assumption that allelic variation in the range of 118–121 cM on chromosome 7H possibly in just one single gene might be responsible for the tocopherol content in the investigated barley genotypes.

**Table 1:** Validation of SNP-markers associated with tocopherol content in two overlapping sets of field-grown barley genotypes.

SNP-marker (i_SCRI_RS_225155) 7H - 120.82 cM			Low-tocopherol SNP-variation: T			p-value	High-tocopherol SNP-variation: C		
year	Tocopherol species	growth conditions	n	Mean-Tph	SE		n	Mean-Tph	SE
2016	gamma	irrigated	34	0.62	±0.05	**0.0267**	22	0.82	±0.08
		dry		0.83	±0.05	**0.0002**		1.54	±0.21
	alpha	irrigated		43.88	±1.91	0.1864		48.37	±2.95
		dry		56.98	±2.16	**0.0052**		69.58	±4.23
2017	gamma	irrigated	33	1.04	±0.12	**0.0002**	25	1.80	±0.14
		dry		4.43	±0.70	**0.0011**		9.95	±1.60
	alpha	irrigated		58.29	±1.67	**<0.0001**		74.19	±2.98
		dry		78.32	±3.29	**<0.0001**		111.30	±5.36
**SNP-marker (i_SCRI_RS_237466) 7**H **- 118.34 cM**			**Low-tocopherol SNP-variation:** **C**			**p-value**	**High-tocopherol SNP-variation:** **T**		
**year**	**Tocopherol species**	**growth conditions**	**n**	**Mean-Tph**	**SE**		**n**	**Mean-Tph**	**SE**
2016	gamma	irrigated	42	0.65	±0.04	**0.0305**	15	0.85	±0.11
		dry		0.94	±0.06	**0.0021**		1.60	±0.30
	alpha	irrigated		42.73	±1.90	**0.0062**		52.75	±2.5
		dry		57.39	±2.01	**0.0015**		73.11	±5.54
2017	gamma	irrigated	40	1.14	±0.12	**0.0002**	19	1.50	±0.18
		dry		6.55	±1.13	0.6903		7.29	±1.23
	alpha	irrigated		58.90	±1.62	**<0.0001**		78.02	±3.00
		dry		84.23	±4.31	**0.0012**		108.50	±4.95
**SNP-marker (i_SCRI_RS_184488) 7**H **- 120.82 cM**			**Low-tocopherol SNP-variation: G**			**p-value**	**High-tocopherol SNP-variation:** **T**		
**year**	**Tocopherol species**	**growth conditions**	**n**	**Mean-Tph**	**SE**		**n**	**Mean-Tph**	**SE**
2016	gamma	irrigated	44	0.64	±0.04	**0.0113**	13	0.89	±0.11
		dry		0.91	±0.05	**<0.0001**		1.80	±0.32
	alpha	irrigated		41.99	±1.75	**<0.0001**		56.80	±2.07
		dry		56.34	±1.96	**<0.0001**		79.07	±4.86
2017	gamma	irrigated	40	1.03	±0.10	**<0.0001**	19	2.17	±0.16
		dry		5.12	±0.90	**0.0037**		10.31	±1.61
	alpha	irrigated		57.63	±1.39	**<0.0001**		80.68	±2.42
		dry		79.96	±3.81	**<0.0001**		117.50	±3.50

The content [ng/mgFW] of γ- and α-tocopherol of flag leaves minus one of barley plants after heading (BBCH-stage 59–77) grown in the rain-out shelter (RS) under well-watered (irrigated) and dry conditions in two consecutive years was analysed by HPLC. Two partially overlapping sets of genotypes were analysed (see material and methods). Three SNP-markers (i_SCRI_RS) for all cultivars were determined by KASP (Competitive Allele Specific PCR) and the mean γ- and α-tocopherol content (Mean-Tph) [ng/mgFW] of all cultivars harbouring each SNP variation is displayed. n: number of genotypes harbouring the SNP variation. SE: Standard error. Significant differences (p < 0.05) are displayed in bold and were calculated by unpaired two-tailed Student’s t-test.

### Evaluation of naturally occurring diversity in *HPT-7H*

**Figure 1:**
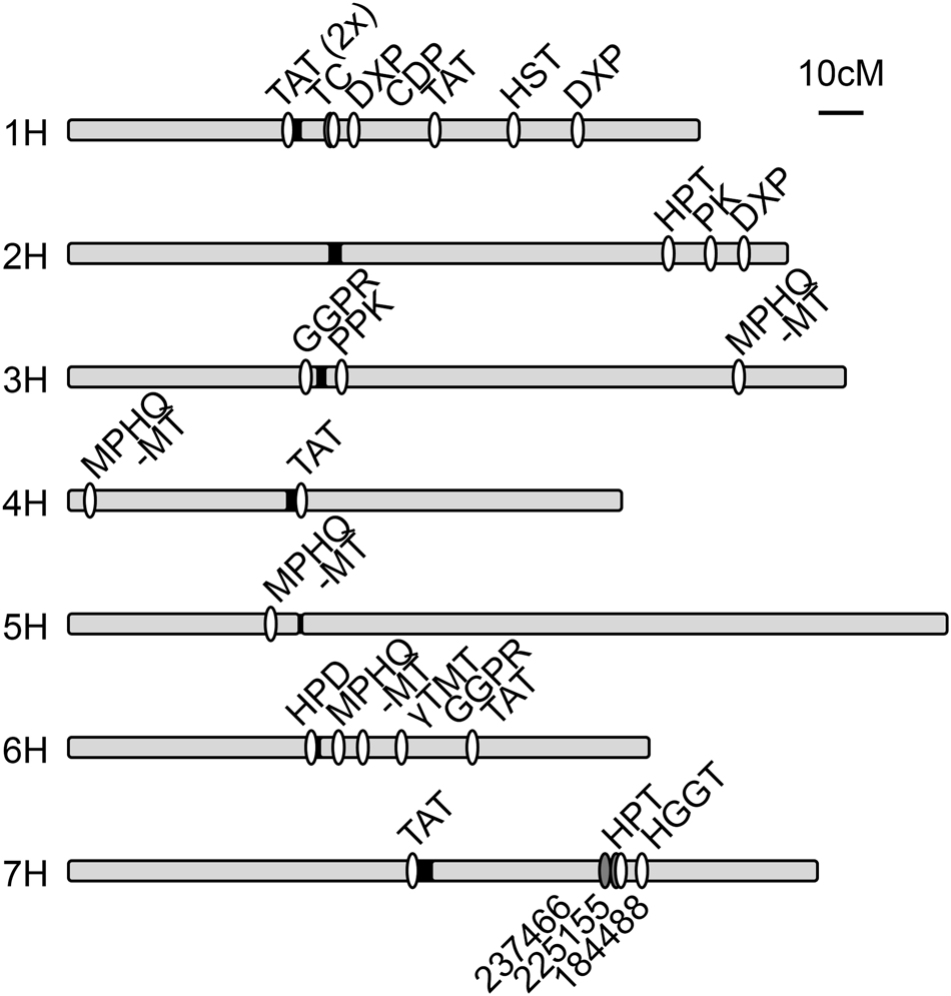
Localisation of structural genes of barley tocochromanol biosynthesis on the barley genetic map. Chromosome arms of the barley chromosomes one (1H) to seven (7H) are displayed as grey bars, centromere regions as black junctions. The positions of the genes (white markings) and SNP-markers (grey markings) are based on map position in centimorgan (cM) according to the IBSC genetic map (see text). γTMT: *γ-tocopherol methyltransferase* (*vte4*); CDP: *chlorophyll dephytylase*; DXP: *1-deoxy-D-xylulose-5-phosphate synthase*; GGPR: *geranylgeranyl diphosphate reductase*; HGGT: *homogentisate geranylgeranyltransferase*; HPD: *4-hydroxyphenylpyruvate dioxygenase*; HPT: *homogentisate phytyltransferase* (*vte2*); HST: *homogentisate solanesyltransferase*; MPHQ-MT: *2-methyl-6-phytyl-1,4-hydroquinone methyltransferase* (*vte3*); PK: *phytyl kinase* (*vte5*); PPK: *phytyl phosphate kinase* (*vte6*); TAT: *tyrosine aminotransferase*, TC: *tocopherol cyclase* (*vte1*). Six-digit numbers: three initially used SNP-markers (as used in Table 1). For biological context see introduction, for further details see Supplementary Table S1.

Exploiting the most recent genome assembly of the International Barley Genome Sequencing Consortium (IBSC, accessible at: http://webblast.ipk-gatersleben.de/barley_ibsc/)^40–42^ we mapped the structural genes of the tocochromanol biosynthesis pathway in the barley genome as well as the position of several genes involved in provision of precursors (Fig. 1). These genes represent prime candidates for the allelic variation underlying the QTL. Previous mapping approaches^38^ were less comprehensive and used different linkage maps^43^ that are not suitable for comparison with our QTLs. Based on its genetic proximity to the investigated SNP-markers, only the *homogentisate phytyltransferase* gene on chromosome 7H (*HPT-7H*) qualified for further investigation (Fig. 1 and Supplementary Table S1). Using stepwise amplification by PCR and subsequent Sanger sequencing we first revised the genomic sequence of the *HPT-7H* alleles present in the three barley cultivars Morex, Barke and Bowman, which allowed to complement the data of the whole genome shotgun sequencing contigs (WGS_contigs) and the Morex reference assembly pseudomolecules (MRA) in the IBSC database. By overlapping multiple reads, we were able to create approximately 6,500 bp long ungapped contigs of all three alleles. They surpassed the one Barke-, the one Bowman- and the two Morex-WGS_contigs in the IBSC database and in addition the newly generated contigs confirmed the orientation and distance of the five contigs of the MRA scaffold thereby closing all existing gaps (Supplementary Fig. S2). Based on tocopherol content and tocopherol pool dynamics under drought stress we choose 12 additional genotypes from our collection and sequenced the genomic region of the *HPT-7H* gene in order to discover additional alleles. Two genotypes carried a fourth allele which we named Umbrella hereafter based on the name of one of those two varieties. We failed to obtain sequence information for the core promotor region 1,000 bp upstream of the known 5’-UTR for this allele even by genome walking. Therefore, the contig of the Umbrella *HPT-7H* allele is only 5,500 bp long. Comparing all four isolated *HPT-7H* alleles, we detected an insertion of a 95 bp fragment between exon one and two in the Umbrella allele and a deletion of 392 bp in the core promotor region 560 bp upstream of the start codon in the Bowman allele (Supplementary Fig. S3). Overall, we identified 55 SNPs or single nucleotide indels in the promotor region and 59 in the transcribed region. At least ten SNPs were found to be specific for each allele and overall seven SNPs resided in the coding region. Two of these SNPs resulted in a K60E and S358N exchange in the primary sequence of the Barke allele compared to the consensus sequence while an A388T exchange in the Bowman allele was evident. The remaining four SNPs located in the open reading frame were silent. Furthermore, five larger allele-specific insertions of 6–12 bp length were identified, with most of them residing in the 3′-UTR (Supplementary Fig. S3 and please see Supplementary Table S4 for compiled allele sequences).

### Identification of *HPT-7H* haplotypes

To determine the *HPT-7H* haplotypes of the remaining genotypes in a fast and cheap manner we developed a CAPS marker (cleaved amplified polymorphic sequence) based identification. The transcribed region of *HPT-7H* harbours three SNPs which display a unique combination in each of the four identified alleles and give rise to the presence or absence of specific restriction enzyme recognition sites (please see Supplementary Fig. S3 and Table S4 for further details). Based on the newly designed CAPS markers we saw that 20 genotypes of our collection carried the Morex-, 43 the Barke-, four the Umbrella- and only one the Bowman-*HPT-7H* haplotype. Acknowledging the potential existence of additional *HPT-7H* alleles that might exhibit identical SNP patterns we will provisionally keep the term haplotype. After comparing the distribution of the identified *HPT-7H* haplotypes with the incidence of the previously employed KASP-markers for the tocopherol QTL we discovered that the genotypes carrying the Morex-*HPT-7H* haplotype predominantly showed those three SNP alleles that were found to be associated with high tocopherol content (Tables 1, 2). Compared to the two other KASP markers the marker i_SCRI_RS_184488 displayed the best match with the *HPT-7H* haplotype distribution which probably can be attributed to its close vicinity to the *HPT-7H* locus: In the MRA scaffold of chromosome 7H this marker (i_SCRI_RS_184488) is located at a distance of approx. 113,000 bp to the *HPT-7H* locus while the others are approx. 1,184,000 bp (i_SCRI_RS_225155) and 5,111,000 bp (i_SCRI_RS_237466) further apart and hence display a weaker genetic linkage to the *HPT-7H* (Supplementary Table S1). The distribution of the row type of the genotypes displays no apparent linkage to the genetic composition (Table 2).

**Table 2:** Distribution of SNP-marker and *HPT-7H* haplotypes in different barley cultivars.

		n	row-type		SNP-marker					
					i_SCRI_RS 225155		i_SCRI_RS 237466		i_SCRI_RS 184488	
					7H - 120.82 cM		7H - 118.34 cM		7H - 120.82 cM	
			two-rowed	six-rowed	low-Tph	high-Tph	low-Tph	high-Tph	low-Tph	high-Tph
					T	C	C	T	G	T
			61	7	38	29	48	20	49	19
Fisher’s exact test:					**p = 0.078**		**p = 1.000**		**p = 0.181**	
row-type	two-rowed	61	61	0	37	24	43	18	46	16
	six-rowed	7	0	7	1	5	5	2	3	4
Fisher’s exact test:			**p = 0.142**		p = 1.837*10 ^−5^		p = 5.692*10 ^−6^		p = 5.967*10 ^−16^	
*HPT-7H* haplotype	Morex	20	16	4	3	17	6	14	1	19
	Barke	43	41	2	31	11	38	5	43	0
	Bowman	1	1	0	1	0	1	0	1	0
	Umbrella	4	3	1	3	1	3	1	4	0

Contingency tables of (1) the row-type of all genotypes to the three initially used SNP-markers (as used in Table 1), (2) the row-type to the *HPT-7H* haplotypes as identified by CAPS-markers, as well as (3) the distribution of the SNP-markers to the *HPT-7H* haplotypes. n: number of genotypes harbouring a particular haplotype, SNP-variation or row-type and the according overlaps. For one six-rowed genotype with the Barke haplotype the marker i_SCRI_RS_225155 was not determined therefore the sum does not fit in the corresponding columns and rows. For all three analysed SNP-markers the high performing variants (always on the right) are primarily found in genotypes carrying the Morex haplotype dark grey cells while the other SNP variants are mainly linked to the Barke haplotype light grey cells. This is especially true for i_SCRI_RS_184488, which is physically more tightly linked to the *HPT-7H* locus than the other two SNPs (Supplementary Table S1). According to Fisher’s exact test, a linkage can be assumed (p < 0.05) between the observed distributions of SNP- and haplotype-variants. In contrast, no linkage of row-type to SNP- or haplotype-variant was detected by Fisher’s exact test (p > 0.05).

### Association of *HPT-7H* haplotypes with the tocopherol pool composition

**Figure 2:**
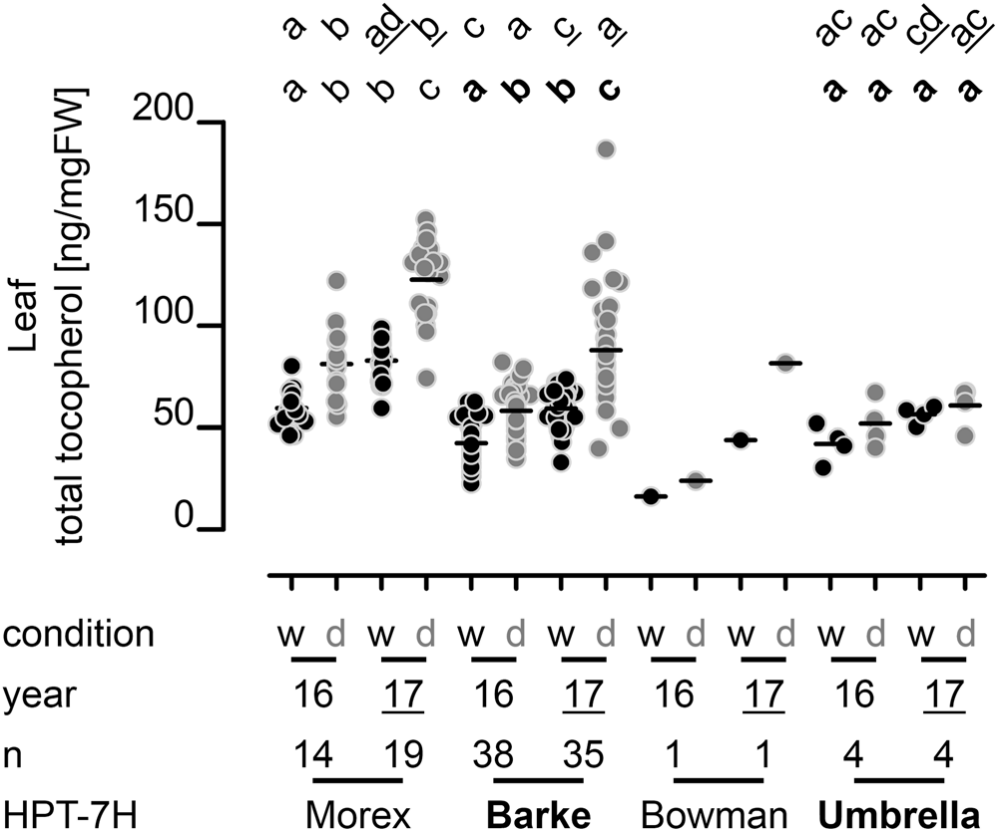
Leaf total tocopherol content of barley genotypes grouped by *HPT-7H* haplotype. The content [ng/mgFW] of total tocopherol (sum of δ-, γ- and α-tocopherol) of flag leaves minus one of barley plants after heading (BBCH-stage 59–77) grown in the rain-out shelter (RS) under well-watered (w – black dots) and dry conditions (d – grey dots) in 2016 (57 genotypes) and 2017 (59 genotypes) was analysed by HPLC. Each dot represents one genotype; genotypes are grouped according to *HPT-7H* haplotypes as determined by CAPS-markers (please see manuscript text and Supplementary Fig. S3). n: number of genotypes harbouring each haplotype. Black horizontal lines: arithmetic means. Significant differences (p < 0.05) are indicated by unequal letters and were calculated in a 1-way analysis of variance (ANOVA) followed by a pairwise Bonferroni post hoc test for all haplotypes in 2016 (top row – regular letters) and in 2017 (top row – underlined letters) as well as for each haplotype covering both years (bottom row – four letters each). For the single genotype with the Bowman haplotype, no ANOVA was possible. Please see Supplementary Fig. S4 for graphs of individual tocopherol subspecies and Supplementary Fig. S5 for leaf tocotrienol content.

After grouping tocopherol contents of all cultivars based on *HPT-7H* haplotypes it was therefore no surprise to find that genotypes with the Morex haplotype had significantly elevated tocopherol contents compared to other genotypes irrespective whether data from different experimental years or from individual growth conditions was regarded (Fig. 2 and Supplementary Fig. S4). In general, plants that were exposed to drought stress accumulated more tocopherol than well-watered plants and in 2017 tocopherol levels in irrigated plants were already as high as the levels in drought stressed plants in 2016. We attribute this phenomenon to elevated temperatures in the second year of the field trials (Supplementary Fig. S6). In the four weeks prior leaf sampling the daily medium temperatures were elevated in average by 2.7 °C in 2017. Particularly higher daily maximum temperatures (ΔTemp^2016/2017^: 6–11 °C) prevailed in the four days preceding sampling in 2017 that affected the plants, to which they apparently reacted by accumulating more tocopherol. The enhanced accumulation of tocopherols in response to drought and elevated temperatures had already been observed in pot-grown barley plants^39^ and is a well-known response of plants (e.g. reviewed in^44^).

### Association of *HPT-7H* haplotypes with inferred population structure

To validate that these observations are not due to a bias caused by overrepresentation of certain genetic backgrounds in the population but are instead more likely to be an attribute of the *HPT-7H* haplotype we investigated the population structure. Based on 4320 SNP markers spread throughout the genome we inferred the population structure using a model-based approach implemented in the software package STRUCTURE. The calculated model proportionally assigns all genotypes to inferred population clusters based on allele frequencies. After estimating a proper population size (Supplementary Fig. S7) we compared the inferred ancestry of the genotypes with the distribution of the *HPT-7H* haplotypes and the row-type of the genotypes (Supplementary Fig. S8). As expected (e.g. as in^45^), six-rowed genotypes displayed high inferred ancestry from one inferred population cluster (here: cluster 1) that is mostly absent in the two-rowed genotypes. In contrast the two-rowed genotypes display high inferred ancestry from the remaining inferred clusters. Despite the strong linkage of certain clusters to row types, linkage of *HPT-7H* haplotypes to any cluster seems to be absent in both years (Supplementary Fig. S8). Therefore, we assume that compared to the row type, which itself had no effect on genetic composition (Table 2), the population structure has no effect on the distribution of *HPT-7H* haplotypes and our observed association of tocopherol content and *HPT-7H* haplotype (Fig. 2 and Supplementary Fig. S4) can be expected to be based on said haplotype.

### *HPT-7H* expression controls tocopherol content

**Figure 3:**
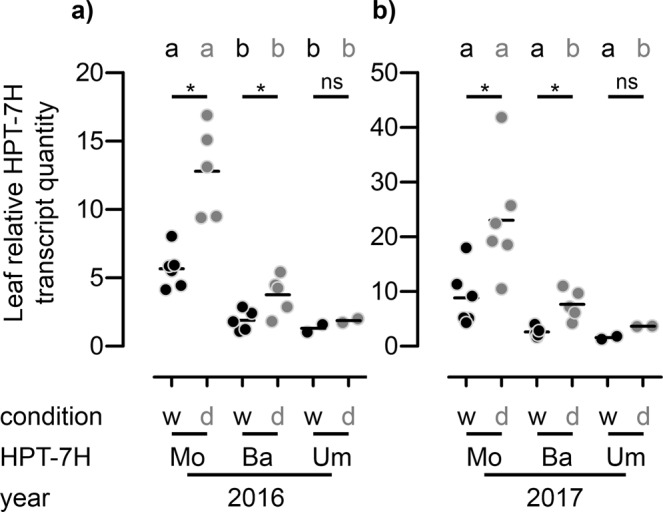
*HPT-7H* transcript level in barley leaves is influenced by the *HPT-7H* allele and is elevated in plants grown under drought conditions. The relative *HPT-7H* transcript quantity of flag leaves minus one of barley plants after heading (BBCH-stage 59–77) grown in the RS under well-watered (w – black dots) and dry conditions (d – grey dots) in **(a)** 2016 and **(b)** 2017 was analysed in both years in the same twelve genotypes by RT-qPCR. Each dot represents one genotype; genotypes are grouped according to *HPT-7H* alleles as determined by Sanger sequencing (Mo: Morex, Ba: Barke, Um, Umbrella). n: number of genotypes harbouring each allele. Black horizontal lines: arithmetic means. Significant differences (p < 0.05) are indicated by an asterisk or unequal letters and were calculated in a 1-way ANOVA followed by a pairwise Bonferroni post hoc test when comparing all three alleles under well-watered (black letters) or dry conditions (grey letters) or for each allele separately comparing both conditions by unpaired two-tailed Student’s t-test.

Having dissected the genetic variability of the *HPT-7H* in our panel of barley genotypes we tried to resolve the molecular basis of the variation in tocopherol content. The Sanger-sequenced Morex and Umbrella alleles code for the same amino acid sequence (see text above) but one allele appears to promote high and the other low foliar tocopherol contents. Therefore, we concluded that differences in transcript levels rather than gene-specific changes in enzyme kinetics are most likely responsible for the observed differences in vitamin E accumulation. We measured the relative transcript quantity of the *HPT-7H* by RT-qPCR in leaves of those 12 genotypes that we had previously used to identify further *HPT-7H* alleles. Since we had sequenced the genomic *HPT-7H* locus in these 12 genotypes completely we can unambiguously distinguish these genotypes by true alleles and not just by CAPS marker-based haplotypes. After grouping the results by allelic composition, we saw that *HPT-7H* transcript levels were highest in genotypes carrying the Morex allele followed by those bearing the Barke and the Umbrella allele (Fig. 3). Furthermore, plants carrying the Morex and Barke allele displayed higher transcript abundance when subjected to drought stress, indicating transcriptional activation of these two alleles under drought stress. This was true for plants grown in 2016 (Fig. 3a) and in 2017 (Fig. 3b).

**Figure 4:**
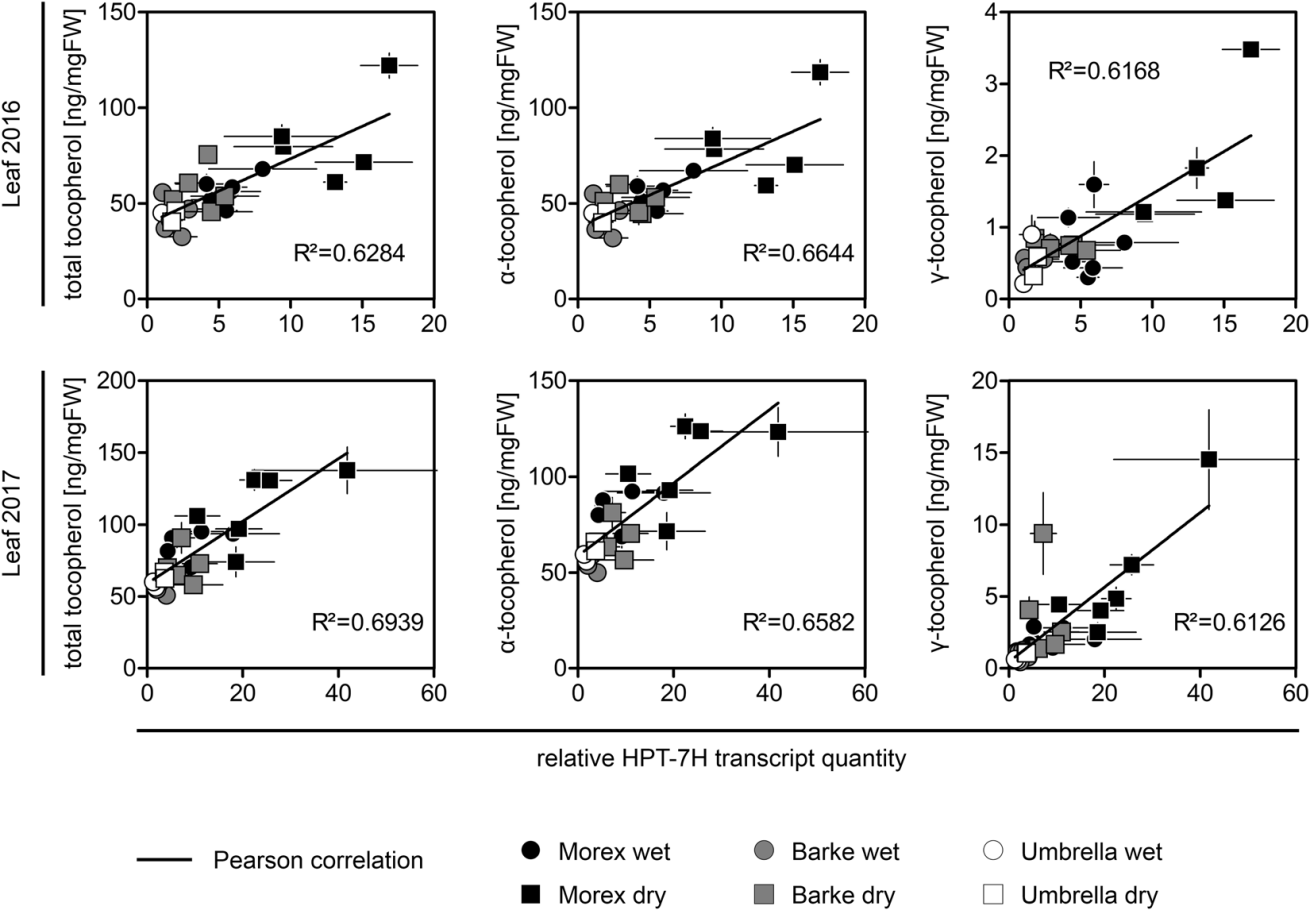
The *HPT-7H* expression level in barley leaves correlates with the tocopherol content. The content [ng/mgFW] of total tocopherol (sum of δ-, γ- and α-tocopherol) (left column), α-tocopherol (middle column) and γ-tocopherol (right column) of flag leaves minus one of barley plants after heading (BBCH-stage 59–77) grown in the RS under well-watered (round data points) and dry conditions (square data points) in 2016 (top row) and 2017 (lower row) was analysed by HPLC and plotted against the relative *HPT-7H* transcript quantity as determined by RT-qPCR. Each data point represents one genotype shaded according to *HPT-7H* alleles as determined by Sanger sequencing (black: Morex, grey: Barke, open: Umbrella). Bars: standard error (n = 4). The black lines indicate the Pearson correlation which was calculated independent of genotype and growth condition. Please see Supplementary Fig. S9 for graphs of individual growth conditions.

Since we had obtained the entire genomic sequence of the *HPT-7H* alleles under study we were curious if differences in basal levels and drought-mediated inducibility can be explained by promoter structure. *In silico* analyses of the *HPT-7H* core promotor region (Supplementary Table S2 - PlantTFDB) revealed a high number of ethylene response transcription factor binding sites in all alleles which explains the general responsiveness of *HPT-7H* to stress conditions (e.g. as reviewed in^46^). Additionally, more MYB and MYB-related binding sites can be found in the Morex allele which might explain the even higher responsiveness to drought stress compared to the other alleles (e.g. as reviewed in^47^). As our sequence of the Umbrella allele lacks large parts of the promotor region and since the Bowman allele contains a large deletion, it is virtually impossible to identify allele specific transcription factor binding sites that affect basal transcription level or *HPT-7H* inducibility based on two alleles (Morex and Barke) only. Therefore, we refrained from drawing any further conclusions from the detected promoter elements and focused on the analysis of the quantitative data. This revealed a close linkage between *HPT-7H* transcript amount and flag leaf minus one tocopherol content. Regarding all genotypes, the content of total-, α- as well as γ-tocopherol was directly proportional to the *HPT-7H* transcript quantity (Fig. 4). This hints to a molecular connection between the amount of the key enzyme and the accumulation of the derived metabolite products. While the content of total- and α-tocopherol correlates well with the *HPT-7H* transcript amount in both years for plants grown under both conditions in the RS, this is not true for the progenitor compound γ-tocopherol which did not show a substantial correlation under well-watered conditions in 2016 (Supplementary Fig. S9). In conclusion, genotypes harbouring the Morex *HPT-7H* allele displayed the highest basal transcript amount of *HPT-7H* as well as the highest foliar tocopherol content in irrigated plants. Under drought conditions *HPT-7H* transcript amounts and tocopherol contents increased correspondingly.

### Tocochromanol content of mature barley grains

**Figure 5:**
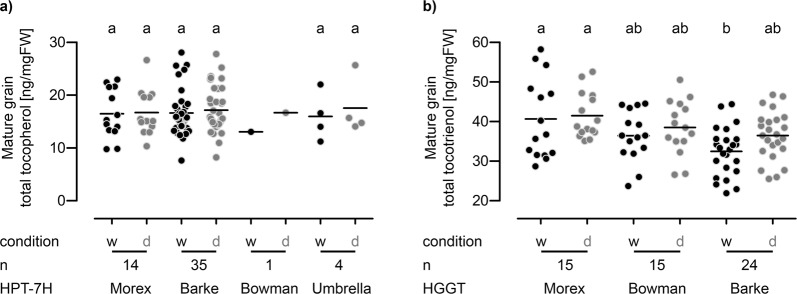
Total tocopherol and total tocotrienol content of mature barley grains. The content [ng/mgFW] of **(a)** total tocopherol (sum of δ-, γ- and α-tocopherol) and **(b)** total tocotrienol (sum of γ- and α-tocotrienol) of mature barley grains of plants grown in the RS under well-watered (w – black dots) and dry conditions (d – grey dots) in 2016 was analysed by HPLC. Each dot represents one genotype; genotypes are grouped according to **(a)** *HPT-7H* haplotypes and **(b)** *HGGT* haplotypes as determined by CAPS-markers (please see manuscript text and Supplementary Figs S3, S10). n: number of genotypes harbouring each haplotype. Black horizontal lines: arithmetic means. Significant differences (p < 0.05) are indicated by unequal letters and were calculated in a 1-way ANOVA followed by a pairwise Bonferroni post hoc test for all haplotypes and conditions in each graph. For the single genotype with the Bowman *HPT-7H* haplotype, no ANOVA was possible. Please see Supplementary Fig. S11 for graphs of individual tocopherol and tocotrienol subspecies.

Given this clear correlation between genetic makeup and tocopherol pool size and composition in leaves we were interested in dissecting the tocochromanol content of mature barley grains. Recently, Graebner and colleagues^38^ identified QTLs for several tocopherol and tocotrienol species in mature grains from a GWAS with 1466 barley accessions. They mapped QTLs for all four tocotrienol subspecies, δ- and γ-tocopherol as well as the content of total tocotrienols and total tocochromanols to positions equivalent to the *HPT-7H* and *HGGT*. The HGGT is the dedicated enzyme involved in tocotrienol biosynthesis^10^ and the *HGGT* gene is located close to the *HPT-7H* on the long arm of chromosome 7H (Fig. 1). This encouraged us to investigate genetic variation of the *HGGT* gene in a similar manner as we did for the *HPT-7H* gene. According to the WGS_contigs the highest genetic variability can be found in the core promotor 1,000 bp upstream of the start codon. We sequenced this area in the 12 genotypes we already analysed before. No additional *HGGT* alleles were found but a CAPS marker could be designed to distinguish between the three *HGGT* haplotypes Morex, Barke and Bowman in the remaining genotypes of the investigated panel (please see Supplementary Fig. S10 and Table S4 for further details). The majority of accessions harbouring the Morex *HPT-7H* haplotype also carried the Morex *HGGT* haplotype. Similarly, the one genotype with the Bowman *HPT-7H* haplotype also harboured the Bowman *HGGT* haplotype. In contrast, the four Umbrella *HPT-7H* haplotypes were either linked to the Morex or the Barke *HGGT* haplotype and the Barke *HPT-7H* haplotype segregated with either the Barke or the Bowman *HGGT* haplotype (Supplementary Table S3). A Fisher’s exact test confirmed that this distribution is very unlikely to be at random (p = 6.278 * 10^−5^). No apparent linkage to a certain population cluster was found for the *HGGT* haplotypes (Supplementary Fig. S8), which is similar to the results obtained for the *HPT-7H* haplotypes. We analysed the tocochromanol content of harvested mature grains from the plants of our first field trial (2016). In line with literature^38,48^ characteristic amounts of tocopherols and tocotrienols could be detected (Fig. 5). The range from low to high vitamin E varieties was less pronounced than in leaves (Fig. 2) and while tocotrienols are underrepresented in green tissues (comprising max. 7% of the total tocochromanol pool) they are the dominant form of vitamin E in mature barley grains contributing to up to 70% of the total tocochromanol content (Supplementary Table S3). Plants grown under drought conditions did neither accumulate significantly more tocopherol nor tocotrienol in their grains compared to plants grown under well-watered-conditions (Fig. 5 and Supplementary Fig. S11), which contrasts the observation made for tocopherols in leaves (Fig. 2). Concerning the total tocopherol content in mature grains no differences could be detected for plants with individual *HPT-7H* haplotypes (Fig. 5a). However, comparing the Barke and Morex *HGGT* haplotypes the latter facilitated a slightly increased content of total-, α- and γ-tocotrienol under well-watered conditions (Fig. 5b and Supplementary Fig. S11) which could reflect the observation by Graebner and colleagues^38^. No clear correlation between tocochromanol content in mature grains and in leaves at heading stage could be detected (Supplementary Table S3).

### Accumulation of tocochromanols in immature barley grains

**Figure 6:**
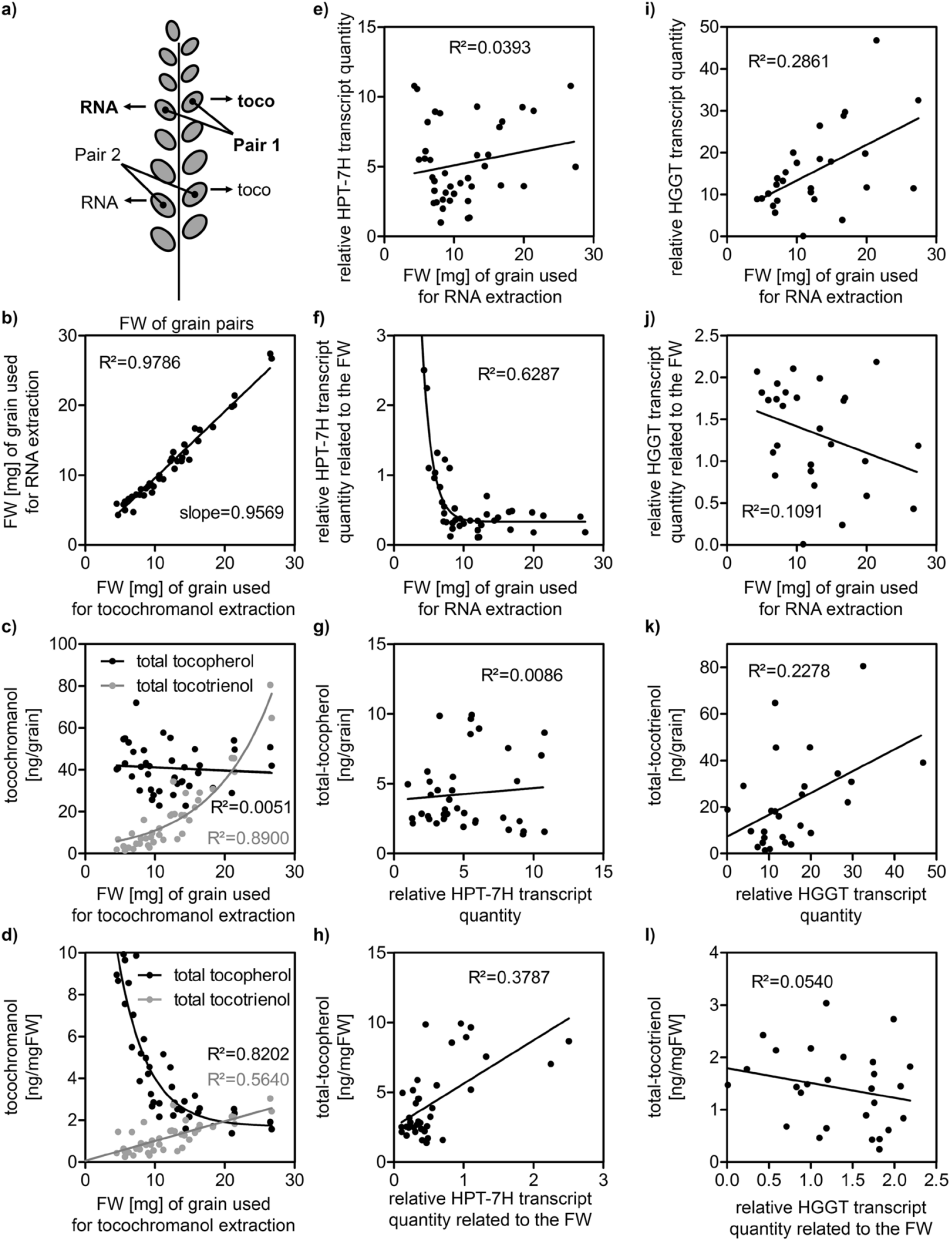
Tocochromanol accumulation and relative quantities of *HPT-7H* and *HGGT* transcripts during seed development. We determined the relative transcript quantities of *HPT-7H* and *HGGT* and the tocochromanol content of immature, still growing grains (milky to early dough stage, BBCH-stage 73–83). Each grain could either be used for RNA or tocochromanol extraction. To be able to relate transcript levels to tocochromanol contents we always used pairs of grains with comparable developmental stage: We chose grains that **(a)** grew close to each other in the ear and had comparable FW as proxy for the age. **(b)** Comparison of the FW of all 40 pairs of grains that were used: X-axis: FW of grained used for tocochromanol extraction; Y-axis: FW of grain used for RNA extraction that was paired with the grain for tocochromanol extraction. Grains from 10 genotypes grown under the two conditions in the RS were analysed. Neither transcript- nor metabolite-level could be related to the growth condition or the haplotypes of *HPT-7H* and *HGGT*. The sole apparent determining factor was the age of the grains estimated by the FW. **(c)** absolute amounts of tocochromanols extractable from each grain [ng/grain] plotted against grain age. **(d)** tocochromanol concentration [ng/mgFW] plotted against grain age. **(e,i)** relative *HPT-7H* and *HGGT* transcript quantities plotted against grain age. **(f,j)** relative *HPT-7H* and *HGGT* transcript quantities in relation to the age of the grains plotted against grain age. **(g,k)** absolute amounts of tocochromanols (as in **(c)**) plotted against the relative *HPT-7H* and *HGGT* transcript quantities (as in **(e,i)**). **(h,l)** tocochromanol concentrations (as in **(d)**) plotted against the relative *HPT-7H* and *HGGT* transcript quantities in relation to the age (as in **(f,j)**). Please see Supplementary Fig. S12 for graphs of individual tocochromanol species.

To assess a potential influence of the *HPT-7H* and *HGGT* expression on vitamin E accumulation in grains we tested immature still growing grains (milky to early dough stage, BBCH-stage 73–83). Since growing grains are transcriptionally and biosynthetically active, we expected a correlation between transcript levels and tocochromanol content. Parallel to leaf sampling in 2017 we harvested developing grains from plants of ten genotypes grown under well-watered and drought conditions in the RS. Each grain was handled individually. Since natural pollination from ear to ear and between genotypes is not synchronized the developmental stages varied between individual plants and genotypes. We tried to obtain a proxy for developmental age by determining the fresh weight (FW) of each grain. As a single grain could only be used for either RNA or tocochromanol extraction pairs of grains with similar FW and growth position in the ear were used to compare the transcript amount of one grain to the metabolite level of the other (Fig. 6a,b).

Apparently, the sole factor determining tocochromanol content was grain FW, which can serve as a proxy for grain age: The total amounts of tocotrienols extractable from each grain ([ng/grain]) increased exponentially over the growth stages covered (Fig. 6c). Putting these amounts in direct relation to the age represented by the weight of each grain ([ng/mgFW]) revealed a linear increase for tocotrienol against FW (Fig. 6d). For tocopherols the absolute amounts extractable from each grain displayed no evident connection to grain age (Fig. 6c). However, an exponential decay of total tocopherol concentrations towards older grains could be observed (Fig. 6d). These seemingly contradictory results are most likely a result of the tissue-specific distribution of tocochromanols inside the grain: By dissecting mature barley grains Falk and colleagues^49^ demonstrated that tocotrienols are predominantly found in the endosperm while tocopherols accumulated in the embryo. Continuous growth of the endosperm and the concomitant shift in favour of endosperm weight might also explain the observed dynamics in relative *HPT-7H* and *HGGT* transcript quantities: The absolute amounts of *HPT-7H* transcripts display no growth-related trend (Fig. 6e) but a steep exponential decay emerges when put in direct relation to grain age (Fig. 6f). Together with tocopherols these transcripts are most likely predominantly extracted from the embryo whose weight contributes less to overall grain weight when grain loading progresses. Notably, a very weak correlation between *HPT-7H* transcript and total tocopherol content was evident when data were normalized to FW i.e. grain age (Fig. 6h). In contrast, the amount of *HGGT* transcript increased with age (Fig. 6i) whereas the data normalized to FW appears unrelated to grain age (Fig. 6j). Together with the tocotrienol products *HGGT* transcripts probably originate mainly from the endosperm whose weight increases relative to overall grain weight in the course of grain loading. Besides the described observations, we found no obvious indication for an altered tocochromanol accumulation in different growth conditions or depending on *HPT-7H* or *HGGT* haplotypes.

## Discussion

### Resolving genetic factors for tocochromanol accumulation in barley

One of the main goals of this study was to analyse the genetic bases of previously identified QTLs for tocochromanol content of barley flag leaves^39^ and grains^38^. By locating structural genes encoding pathway enzymes involved in vitamin E biosynthesis in the barley genome assembly (Fig. 1) we could shortcut the route to identify two casual genes that underlie these metabolic QTLs and were able to avoid fine mapping of the chromosomal regions. One of the four uncovered *HPT-7H* alleles and one of the three uncovered *HGGT* alleles can be classified as a high performing allele (Figs 2, 5) and can now be exploited for marker assisted breeding of high vitamin E barley varieties. This emphasizes the power of genome wide association studies and mapping of metabolic QTLs for plant breeding efforts, especially when combined with candidate gene-based approaches as described in this publication, because this further enhances the controllability of the desired trait due to omission of recombination events. We identified the transcript level of *HPT-7H* to be a crucial factor for foliar tocopherol accumulation (Fig. 4). Transcript levels of structural genes of the vitamin E biosynthetic pathways are well-known determinants of tocochromanol levels in other plant systems as proven by overexpression, knockdown or knockout lines (extensively reviewed in^25^). Similarly, an increase of tocochromanol levels in abiotic stress-conditions has also been reported several times for various plant species (e.g. reviewed in^50^). In contrast, the influence of environmental- or developmental-changes on transcript levels of structural genes of the vitamin E pathway has been studied less frequently. In context of senescence, jasmonate was shown to elevate mRNA levels of an *Arabidopsis TAT* causing an accumulation of γ-tocopherol^51^. After applying methyl jasmonate and ethylene to barley leaves or after inducing oxidative damage Falk and colleagues^52^ observed an increase of barley *HPD* mRNA levels and Singh and colleagues^53^ could directly show that the ethylene inducibility of the mango *HPD* resulted in increased foliar tocopherol levels. GWAS and RNAseq studies were combined in maize grown devoid of stress conditions by Diepenbrock and colleagues^20^ who concluded that genotype-specific differences in mRNA levels of vitamin E affiliated genes that map to some QTLs determine the tocochromanol composition. Further genotype-specific differences of transcript levels for structural genes of the tocopherol pathway were discovered in tomato introgression lines. By dissecting a previously identified QTL for the tocopherol content of ripe tomato fruits^27^ Quadrana and colleagues^54^ identified two epialleles of a *VTE3* gene derived from a domesticated (*Solanum lycopersicum*) and a wild tomato variety (*Solanum pennellii*) which confered different tocopherol levels by different *VTE3* transcript levels. Similar, ten haplotypes of a *yTMT* where identified in 137 rice accessions^37^. After defining two phylogenetic groups based on the promotor sequence Wang and colleagues^37^ showed that enhanced *yTMT* transcript levels in one group are linked to elevated α-tocopherol contents^37^. In summary, Quadrana and colleagues^54^ and Wang and colleagues^37^ were able to decipher allelic variation of tocopherol biosynthetic genes and link these variations to gene expression. They also established that elevated expression of these genes resulted in elevated tocopherol levels in tomato and rice, respectively. Here we show allelic variation of tocopherol biosynthetic genes in barley and identified alleles resulting in different levels of expression and tocopherol content. Unlike previous studies we were able to demonstrate that increased stress-inducibility of individual *HPT-7H* alleles is the basis for improved tocopherol accumulation in barley leaves during drought stress under field conditions (Figs 2, 3, 4).

### Vitamin E accumulation to enhance plant fitness

In leaves, tocopherols prevent oxidative damage of thylakoid membranes and hence allow efficient photosynthetic electron transport. Consequently, it has been reported that vitamin E can delay leaf senescence^55,56^ and thereby potentially enable longer phases of photosynthesis and grain filling. In this context many reports attribute a beneficial effect of tocopherol accumulation on plant fitness as it protects plants against abiotic stress conditions such as salinity, drought, heat, metal toxicity, ozone and UV radiation (e.g. extensively reviewed in^44^). In case of barley, Templer and colleagues^39^ observed a positive correlation between leaf tocopherol content and drought tolerance of pot-grown barley plants. Based on these previous studies we speculated that tocopherol accumulation should enhance stress tolerance of field grown barley plants. To validate this assumption, we analysed a broad range of barley accessions with different basal tocopherol levels in leaves and grains. Unfortunately, we were not able to resolve a clear positive correlation between tocopherol content and plant performance under drought stress. This obvious discrepancy might be explained by short-term and long-term responses of plants to adverse environmental conditions. Vitamin E accumulation might be more beneficial during short-term adaption to rapidly emerging stress as seen in pot-grown plants upon abrupt water withdrawal of previously non-stressed plants^39^. Munné-Bosch^50^ already reported that tocopherol accumulation follows a biphasic pattern in which an initial increase can be followed by a drastic decline in tocopherol pool size. The gradual decline of water availability in the silty clay soil in the RS (Supplementary Fig. S1) might be unsuited to resolve beneficial short-term effects of tocopherols on plant fitness. Furthermore, it is known that plants can compensate the lack of tocopherols by increased levels of ascorbate^50^ and in general plant adaptation to stress conditions include a variety of strategies, such as altered leaf architecture, altered stomata responsiveness, accumulation of compatible solutes, antioxidants or ROS scavenging capacity. The artificial alteration of some of these mechanisms by biotechnological means can improve drought tolerance (e.g. reviewed in^57–59^). In our field trails, we reproducibly observed a stress-mediated increase of tocopherol content in barley leaves in two consecutive years and the accumulation of tocopherol always correlated with the severeness of the stress applied (Supplementary Fig. S13). We therefore assume this behaviour implies a role of tocopherol in stress adaptation.

### Different tocochromanol distribution in barley grains

Interestingly, γ-tocopherol as the penultimate pathway product increases more strongly than the end product α-tocopherol when *HPT-7H* gets induced in stressed barley leaves (Figs 3, 4 and Supplementary Fig. S13). This suggests that enhancing metabolic flux at the starting point of the pathway causes a bottleneck in the downstream conversion from γ- to α-tocopherol limited by the enzymatic activity of γTMT. Comparable observations have been made in transgenic *Arabidopsis thaliana* plants. Artificial overexpression of *AtHPT* increased the content of total tocopherol in leaves but also shifted the pool composition in favour of γ-tocopherol^60^. Simultaneous overexpression of *AtγTMT* restored the ratio of α- to γ-tocopherol in leaves^60^. In *Arabidopsis* seeds γ-tocopherol is the major form to accumulate which in turn can be artificially altered by overexpression of *AtγTMT*. This enhances the metabolic flux towards α-tocopherol^60^, demonstrating that enzymatic γTMT activity is strongly limiting in wild type seeds^61^. In mature barley grains 20% of the total tocopherol pool and 40% of the total tocotrienol pool are comprised of their respective γ-tocochromanol isoform (Supplementary Fig. S11). This also indicates limiting γTMT activity in barley grains and the pronounced abundance of the γ-isoforms seem to be characteristic for mature grains of cereals like rye, wheat, oat, spelt, rice and barley^48,62^. However, these ratios do not prevail throughout grain development as the shares of the γ-isoforms experience strong changes in younger grains (Supplementary Fig. S12). Falk and colleagues^49^ demonstrated that tocopherols predominantly accumulate in the embryo, while tocotrienols reside in the endosperm of barley grains. Comparable distributions are known for oat^63,64^ and rice^65^ and we assume that our metabolite- and transcript data of immature grains reflect these distributions as the contribution of the embryo weight to total grain weight decreases with grain development. At the developmental stages covered in our samples we did not see a correlation of metabolite content and transcript level of *HPT-7H* and *HGGT*, which would emphasize the key regulatory role of those genes for the respective tocochromanol content (Fig. 6g,h,k,l). The weak correlation between *HPT-7H* transcript levels and tocopherol contents at different stages of grain development (Fig. 6h) only emphasize the presumed parallel extraction from the embryo. Both parameters decline simultaneously to the same degree, as embryo size decreases relative to the endosperm. Furthermore, no response of transcript level or metabolite content to drought stress was evident in neither mature nor immature grains (Figs 5, 6) which can be explained in two ways. Either tocochromanol biosynthesis possesses a different transcriptional responsiveness to drought in grains compared to leaves (Figs 2, 3), or the plants protect their reproductive tissues effectively from water shortage. While plant biomass and total yield of grains were heavily affected by drought stress in the RS, parameters that are indicators for the quality of the grains like grain sorting, thousand grain weight (TGW), extract and protein content were least affected (Supplementary Fig. S14). This hints towards an effective protection of grains: Even though grain yield was reduced, the quality of each grain was not affected by drought, which may indicate that individual grains did not experience severe stress and hence no induction of vitamin E biosynthesis would be detectable. In mature grains the Morex *HGGT* haplotype conferred slightly increased levels of tocotrienols over the Barke *HGGT* haplotype (Fig. 5), which might be the genetic bases of the QTL for grain tocochromanol content identified by Graebner and colleagues^38^. The difference of high to low tocotrienol content in mature grains was significant but not as pronounced as the difference between high and low tocopherol content in leaves (Figs 2, 5). Since no connection between *HPT-7H* haplotype and tocopherol content was observed in mature grains (Fig. 5) *HPT-7H* and *HGGT* transcript levels could not accounted for the tocochromanol content of immature grains (Fig. 6). Probably, vitamin E accumulation presumably depends on additional factors in barley grains which have yet to be uncovered. Obvious candidates are genes that map to positions of other QTLs that are associated with grain tocochromanol content^38^. The only QTL that maps to a gene identified in the frame of this study (Fig. 1) links the content of γ-tocotrienol close to the map position of the *γTMT* on chromosome 6H^38^. As judged by the WGS_contigs of the cultivars Morex, Barke and Bowman, *γTMT* displays less allelic diversity compared to *HPT-7H* and *HGGT* and was therefore deemed less suitable to be exploitable for the creation of genomic markers for tocochromanol content in the beginning of this study.

### The relevance of increasing vitamin E content in feed and food

Genetic engineering of biosynthetic pathways was regarded as a powerful tool to improve human health since the early days^66^. As vitamins are, by definition, crucial for the mammalian diet, high vitamin E accessions should be relevant for commercial applications. While elevated contents of tocopherol in green tissues of cereals are hardly relevant for human diet, they are relevant for livestock feed. According to Newman and Newman^67^ and Zhou^68^ over 60% of the produced barley biomass are relegated to feed as hay or as straw after threshing. Uptake of vitamin E into the mammalian bloodstream is facilitated by the α-tocopherol transfer protein in the liver^69^, which displays the highest affinity towards the eponymous α-tocopherol and represents the limiting factor for vitamin E activity *in vivo*^70^. The elevated levels of α-tocopherol in leaves facilitated by the Morex *HPT-7H* haplotype (Supplementary Fig. S4) can be considered relevant for the quality of hay and straw. The elevated contents of tocotrienols in mature grains (Fig. 5 and Supplementary Fig. S11) confered by the Morex *HGGT* haplotype can be considered relevant for the fitness of the grains. The fundamental fitness of each grain can be regarded as its ability to germinate, which is controlled by the tocochromanol content: vitamin E deficient grains and seeds of transgenic rice^71^ and *Arabidopsis* plants^72^ displayed a drastic decrease in longevity as they are less resilient towards oxidative damage during dormancy. High germination rates are of paramount interest for farmers and uniform germination properties represent an important feature of high quality malt for brewing^73^ for which about 30% of the produced barley biomass are used^67,68^. Furthermore, higher grain vitamin E contents were reported to result in higher contents in processed malt^74^. This can have an additional positive influence on the brewing process and its product: As demonstrated by Zhang and colleagues^75^ brewing yeasts display enhanced viability during the fermentation process in vitamin E rich conditions and therefore can tolerate and produce higher levels of ethanol.

## Conclusion

We were able to validate previously mapped QTLs for the vitamin E content of barley leaves and grains. The genetic bases of these QTLs were unravelled by identifying alleles of two genes involved in vitamin E biosynthesis, *HPT-7H* and *HGGT* on barley chromosome 7H. The alleles differ in their capacity to drive vitamin E accumulation, more specifically, transcript quantity of the *HPT-7H* was revealed as the pace-maker of tocopherol biosynthesis in leaves. The *HPT-7H* mRNA level differed between identified *HPT-7H* alleles and was shown to be inducible in stressed leaves, linking the genetic diversity of this gene to the foliar tocopherol accumulation behaviour of barley plants in response to stress conditions. The gained insights emphasize the power of genome wide association studies and mapping of metabolic QTLs for plant breeding efforts and will assist in the creation of new barley cultivars.

## Material and Methods

### Plant material and growth conditions

Two sets of genetically diverse spring barley genotypes (Supplementary Table S3) were grown under a mobile rain-out shelter on tracks (RS) at the Bavarian State Research Center for Agriculture (LfL) (Freising, Germany). The first set of 57 genotypes was grown from 05^th^ April 2016. The second set of 59 partially overlapping genotypes was grown from 30^th^ March 2017. To ensure even distribution of moisture in the silty clay soil at the beginning of the experiments the RS shielded the experimental area from natural precipitation for about four months before sowing. After sowing the RS was programmed to only move over the experimental area during rain. This shielded the plants and controlled watering regimes by artificial irrigation could be established. The experimental area was divided into four plots. Each genotype was grown once in each plot in a double row of 40 individual plants. The position of each genotype in each plot was randomized. Two plots diagonal from each other were artificially irrigated from above with 20 mm/m² water once or twice a week (Supplementary Fig. S1) to create well-watered conditions while the other two quarters did not receive any water. Drought conditions were tightly monitored by tensiometer measurements in 15 cm, 45 cm and 75 cm depth across the entire plot. At the time of leaf sampling substantial drought stress with soil moisture below the wilting point (−500 hPa) had prevailed in 45 cm depth for at least ten days in the non-irrigated plots (Supplementary Fig. S1). Temperatures were logged at a nearby (approximate 300 m) weather station (Supplementary Fig. S6).

### Leaf sampling for metabolite measurements and gene expression analyses

Fully expanded flag leaves minus one of barley plants grown in the RS (BBCH-stage 59–77) were sampled. From the harvested leaves 1–2 cm from the base and 3–5 cm from the tip were removed prior to deep-freezing in liquid nitrogen and long-term storage at −80 °C. Four sample pools comprising four to six leaves were generated for each genotype and condition. Due to the large sample number (>450) a longer sampling period from late morning to late afternoon could not be avoided. To distribute daytime effects as good as possible replicate one for each genotype grown under well-watered conditions was taken before replicate one for each genotype under drought conditions, followed by replicate two for well-watered conditions for each genotype and replicate two for drought conditions and so on.

### Sampling of grains

At the same day leaf samples were taken in 2017 immature grains of ten selected genotypes were harvested. Per genotype and condition one representative ear was chosen that still had grains in the early to late milk stage (BBCH-stage 73–77). Starting from the bottom of the ear each grain was removed and frozen separately leaving the husks in the ear. The fresh weight (FW) of all grains was determined afterwards to find pairs of grains grown at similar position and with similar FW (Fig. 6a,b). Mature grains of 2016 were analysed after harvest.

### Quantification of tocochromanols

The tocochromanols of leaf samples (40–80 mgFW) were extracted as described in^76^. Tocochromanols of mature barley grains were extracted as adapted from the methods of^48,76^: ten grains were ground using a household MC 23200 coffee grinder (Siemens, Munich, Germany) and aliquots (80–100 mgFW) of this fine powder were used for extraction. Successively four times the powder was extracted with 1 ml 100% methanol for 1 h at 4 °C at constant horizontal shaking at 1,500 rpm. To prevent sedimentation of the powder during shaking stainless steel beads were added to the reaction tubes. The supernatants were pooled accordingly, concentrated using a vacuum concentrator and suspended in 250 µl methanol and stored on ice. We combined both methods to extract tocochromanols from immature grains: entire grains were ground individually in liquid nitrogen and extracted three times with the horizontal shaking method with stainless steel beads. The tocochromanols in the final extracts were detected and quantified with a Dionex Ultimate 3000 HPLC system (ThermoFisher Scientific, Schwerte, Germany) system as described in^76^.

### DNA extraction

For genotyping genomic DNA was extracted from barley leaf tissue using an adapted protocol^77^ scaled down by a factor of 100. Additionally, the DNA was treated with 0.4 µl RNAse (10 µg/µl) in 500 µl 10:1 TE (Tris-HCl/EDTA) puffer (pH 7.5) for 1 h at 60 °C followed by a precipitation step with 500 µl Chloroform:Isoamyl alcohol (CI, 24:1 v/v) at 8,000 g for 10 min. DNA was then precipitated from the aqueous phase by addition of 50 µl 3 M Sodium acetate (pH 5.2) and 2 volumes 100% ethanol, incubation for 1 h at −20 °C and centrifugation for 20 min at 17,000 g. It was washed twice with 1 ml 70% ethanol, dried and suspended in 50 µl H_2_O bidest. and stored at −20 °C until further use.

### PCR, Sanger sequencing & CAPS-marker

Amplification of genomic fragments of *HPT-7H* and *HGGT* was performed with Phusion® High-Fidelity DNA Polymerase (New England Biolabs, Frankfurt am Main, Germany) according to the manufacturer’s guidelines. Primers (Supplementary Table S4) were synthesised by metabion (Planegg/Steinkirchen, Germany). Sanger sequencing was performed by Eurofins GATC Biotech (Konstanz, Germany). Evaluation and interpretation of the sequences was done with the Geneious software^78^. Digestion of PCR-fragments for CAPS markers were performed according to the manufacturer’s guidelines provided with the restriction enzymes. Please see Supplementary Table S4 for compiled allele sequences.

### KASP-marker

Allele-specific primers for the SNP markers i_SCRI_RS_225155, i_SCRI_RS_237466, and i_SCRI_RS_184488^79^ (and as already used in^39^) were designed by LGC (Teddington, UK) and supplied as a primer mix which could directly be used in the KASP reaction. 3 µl 2 × KASP PCR mix (also LGC), 3 µl genomic DNA template (10 ng/µl) and 0.084 µl primer mix were incorporated into the KASP reaction. Amplification was performed in a Light Cycler® 480 instrument II (Roche, Basel, Switzerland), starting with 15 min at 94 °C, a touchdown phase of 10 cycles at 94 °C for 20 s and at 61 °C for 60 s with a 0.6 °C decrease in temperature per cycle, followed by 26 cycles of 94 °C for 20 s and 55 °C for 60 s. After the initial PCR reactions a recycling step was included with 94 °C for 20 s followed by an annealing/elongation step at 57 °C for 60 s. The fluorescence signals were acquired at 520 nm and 556 nm for 2 min at 25 °C.

### Genotyping and analysis of population structure

All barley genotypes investigated in this study but one were genotyped by Traitgenetics GmbH (Gatersleben, Germany) with an Illumina iSelect 9 K SNP array containing 7864 genome-wide single nucleotide polymorphism (SNP) markers^80^ that already had been used in our previous study^39^. Population structure was investigated independently for each genotype set (2016, 2017). To this end 4320 SNPs were chosen that had a minor allele frequency of at least 10% and maximum missing data frequency of 10% in both sets simultaneously. We choose a model based approach implemented in the software package STRUCTURE v2.3.4^81–84^ to infer population structure. We choose ploidy of data = 2 to take heterozygosity into account, used the admixture model, no prior population information and correlated allele frequencies. As suggested in the documentation of the program when using sets of SNP-data it can be favourable to estimate λ (allele frequency distribution) first by running the program at *K = *1 (number of populations) and then fix it for subsequent runs. This was done five times for both sets and λ was fixed at the mean of these runs as following (2016: 3.13606 (±0.01373) and 2017: 2.86704 (±0.02380)). All remaining parameters were kept at the default settings. Then for each *K* from 1 to 15 the population structure was inferred 20 times. All runs were performed using a burn-in length of 100,000 iterations followed by 500,000 MCMC (Markov Chain Monte Carlo) iterations. Based on the posterior probability of *K* (ln Pr(*X*|*K*)) provided by the program after each run as a parameter called *estimated ln prob of data*, we choose *K* = 5 (2016) and *K* = 6 (2017) as they plateau at these values stable. Additionally, we looked at the Δ*K* values as suggested by Evanno and colleagues^85^. Those values display maxima at the chosen *K* values and certify the choice (Supplementary Fig. S7 and Table S6). For further contemplations the inferred ancestry of individuals (the estimated membership coefficients for each individual in each cluster) of all 20 runs of the chosen number of populations (*K*) was compiled (Supplementary Table S3) for displaying (Supplementary Fig. S8) in this study.

### RNA extraction

For expression profiling total RNA was extracted from approximately 50 mg fresh barley leaf tissue or single immature grains using a guanidinium thiocyanate (GITC)-based protocol adapted from the method of Chomczynski and Sacchi^86^. Quickly, leaf material or grains were ground in liquid nitrogen cooled mortars and homogenised in 800 µl GITC-containing solution (4 M GITC, 25 mM Sodium citrate, 0.5% (w/v) N-Lauroylsarcosine sodium salt, pH 7.0 (NaOH)). The homogenate was transferred to a 2 ml reaction tube, 1/10 volume (80 µl) 0.2 M sodium acetate (pH 4.0) was added and vortexed briefly to extract DNA. One volume phenol was added, vortexed vigorously and incubated on ice for 30 min. ¼ volume CI was added, vortexed vigorously and incubated on ice for 15 min. Phases were separated by spinning in a benchtop centrifuge at 6,000 g for at least 10 min. The aqueous phase was transferred to a fresh 2 ml reaction tube and organic compounds were extracted with 800 µl PCI (Phenol:Chloroform:Isoamyl alcohol (25:24:1 v/v)) after a 10 min incubation on ice and subsequent centrifugation at 6,000 g for at least 10 min. This PCI step was repeated once. In the end total RNA was precipitated from the aqueous phase be adding 1/10 volume 1 M acetic acid and 1 volume 100% ethanol. After incubation for at least 15 min at −20 °C RNA was precipitated by full speed centrifugation in a benchtop centrifuge for at least 20 min. The RNA pellet was washed once with 800 µl 3 M NaOAc (pH 6.0) and twice with 800 µl 80% ethanol (v/v) at full speed for at least 20 min each. Residual ethanol was let evaporate and the RNA dissolved in 50 µl DEPC-treated water. DEPC-treated water and the 3 M NaOAc solution were prepared as follows: 0.1% (v/v) Diethyl pyrocarbonate (DEPC) was added to H_2_O bidest. or 3 M NaOAc, mixed well and kept at room temperature overnight. Afterwards the solutions were autoclaved twice to heat denature the DEPC. DEPC-treated water was also used to dilute 100% to 80% (v/v) ethanol. RNA purity and integrity were verified with a NanoDrop ND-1000 (ThermoFisher Scientific, Schwerte, Germany) and an Agilent 2100 Bioanalyzer (Agilent Technologies, Frankfurt am Main, Germany).

### RT-qPCR

A total of 1 µg leaf or 100 ng grain total RNA were used for cDNA synthesis using RevertAid H Minus Reverse Transcriptase (ThermoFisher Scientific, Schwerte, Germany) according to the manufacturer’s guidelines. Relative transcript quantities of *HPT-7H* and *HGGT* were determined in technical triplicates using an Agilent Technologies G8830A AriaMx Real-Time PCR machine (Agilent Technologies, Frankfurt am Main, Germany) in combination with Agilent Technologies Brilliant III Ultra-Fast SYBR® Green QPCR Master Mix (Agilent Technologies, Frankfurt am Main, Germany) according to the manufacturer’s guidelines using the *HvActin3* transcript for normalization. Please see Supplementary Table S4 for oligonucleotide primer sequences used.

### Statistical analyses

Fisher’s exact test was performed using the statistical language R (version: 3.5.1, R Core Team 2018). All remaining statistical analyses were performed with GraphPad Prism 5 software as stated in each figure legend.

## Supplementary information

Supplementary Figures

Supplementary Table S1

Supplementary Table S2

Supplementary Table S3

Supplementary Table S4

Supplementary Table S5

Supplementary Table S6

## Data Availability

All data generated or analysed during this study are included in this published article and its Supplementary Information files.
