# Supplementary material for: Deciphering the genetic basis for vitamin E accumulation in leaves and grains of different barley accessions
Source: Sci Rep. 2019 Jul 1;9:9470. doi: 10.1038/s41598-019-45572-7 (PMC6602966; doi:10.1038/s41598-019-45572-7)
Supplement: Supplementary file 1 — Supplementary Figures [file 41598_2019_45572_MOESM1_ESM.docx]

**Supplementary Figures**

Deciphering the genetic basis for vitamin E accumulation in leaves and grains of different barley accessions

Christian Schuy*, Jennifer Groth, Alexandra Ammon, Julia Eydam, Steffen Baier, Günther Schweizer, Anja Hanemann, Markus Herz, Lars M. Voll, Uwe Sonnewald

*Correspondence: christian.schuy@fau.de +499131-8528261 https://orcid.org/0000-0002-3104-3551

**
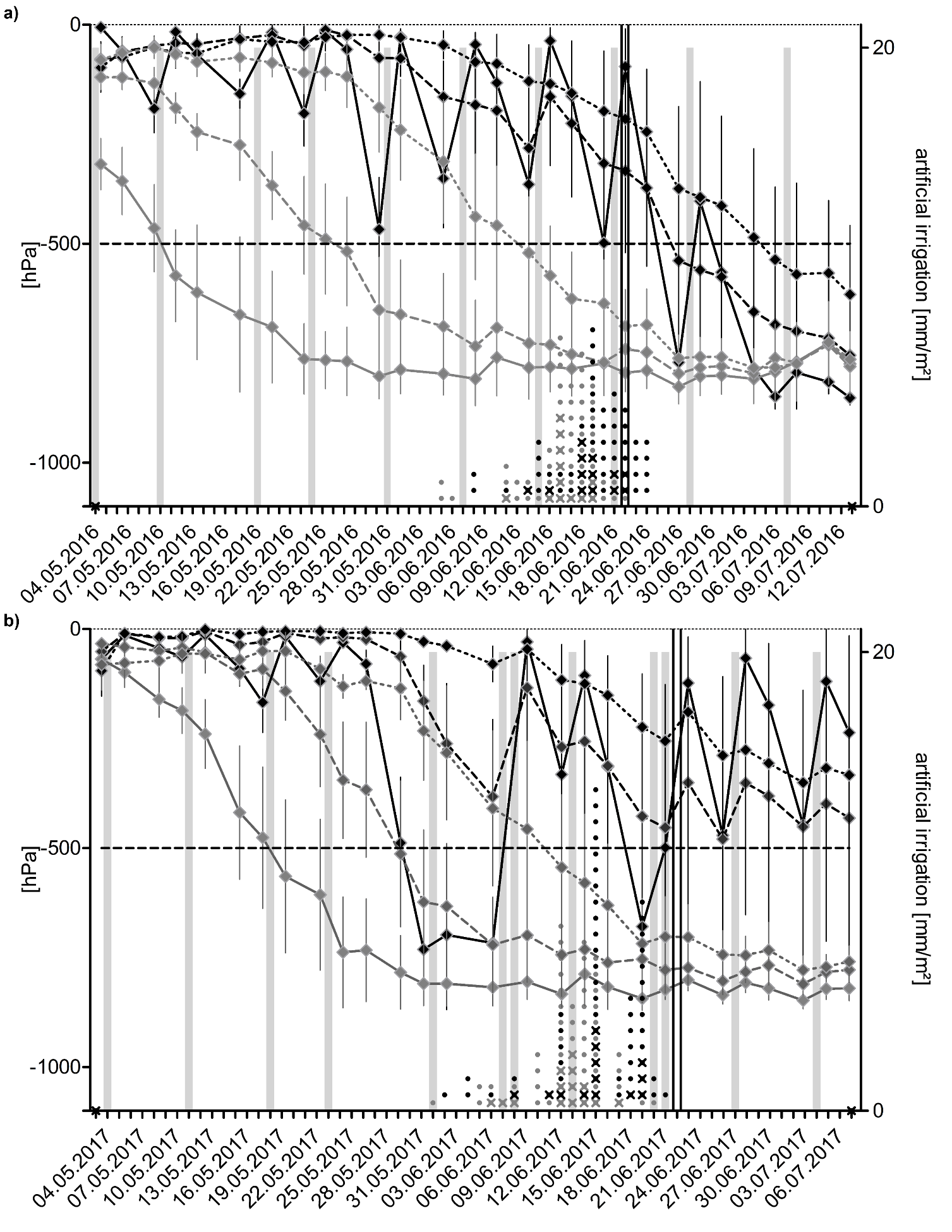
**

**Fig. S1: Soil moisture data, irrigation schedule and heading dates from the 2016 and 2017 rain-out shelter experiments**

Courses with thin vertical error bars: Soil moisture (soil moisture tension [hPa]) was determined 2-3 times a week throughout the experimental area by 8 tensiometers for well-watered (black lines) and dry conditions (grey lines) in 15 cm (solid lines), 45 cm (dashed lines) and 70 cm depth (dotted lines). Well-watered/irrigated plots were watered 1-2 times a week from above with 20 mm/m² at the dates indicated by thick grey vertical bars. In both years samples were taken on the 22^nd^ of June (Two black parallel lines). Heading dates for each individual genotype are indicated in a histogram for irrigated (black dots) and for drought stressed plants (grey dots). Those genotypes additionally labelled by a cross where later chosen for expression profiling of leaf material (**Fig. 3 + 4** and Supplementary **Fig. S9**). At time of heading drought stress (-500 hPa) was achieved for all genotypes in both years in at least 45 cm depth, a common depth for barley roots. For reference: Dates of sowing: 05.04.2016 and 30.03.2017. Please see Supplementary **Tab. S3** online for heading dates and Supplementary **Tab. S5** online for tensiometer raw data.


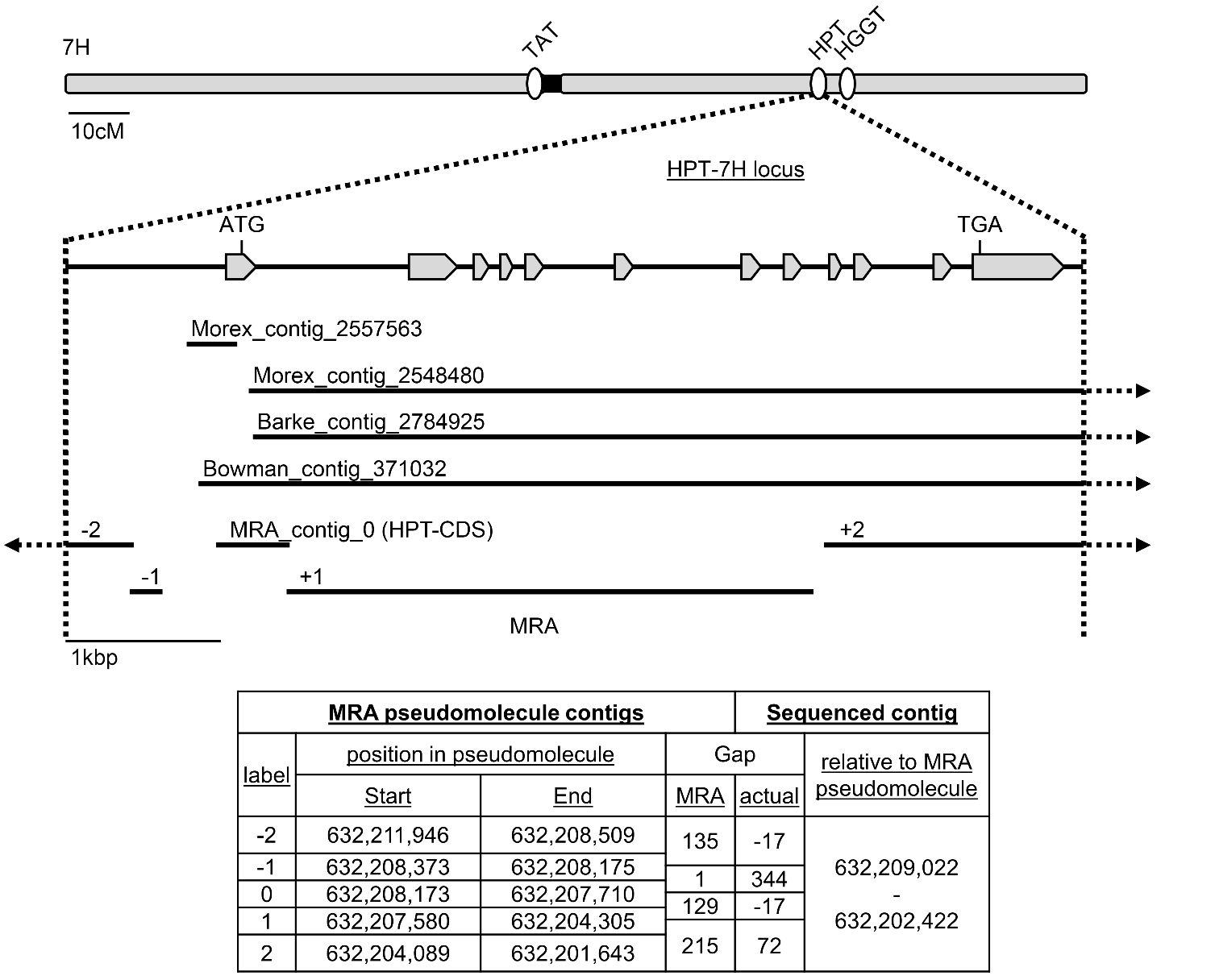


**Fig. S2: Genomic locus of the barley *HPT* on chromosome 7H as determined by Sanger sequencing**

Chromosome arms of the barley chromosome 7H are displayed as grey bars, the centromere region as black junction. The positions of the genes are based on map position in centimorgan (cM) according to the IBSC genetic map (see text). Grey bars in the indicated *HPT-7H* locus represent exons based on cDNA information. The genomic locus of the *HPT-7H* as it is depicted here was determined by stepwise PCR-amplification followed by Sanger-sequencing. The genomic sequence information of the WGS_contigs of the three barley cultivars Morex, Barke and Bowman as well as the pseudomolecule 7H of the Morex reference assembly (MRA) were used as templates. The position of each contig in the pseudomolecule 7H is given in base pairs (bp) as well as the supposed (IBSC genome data) and the actual gap as determined by Sanger sequencing. Negative gaps represent overlaps. Please see Supplementary **Tab. S4** online for compiled allele sequences.


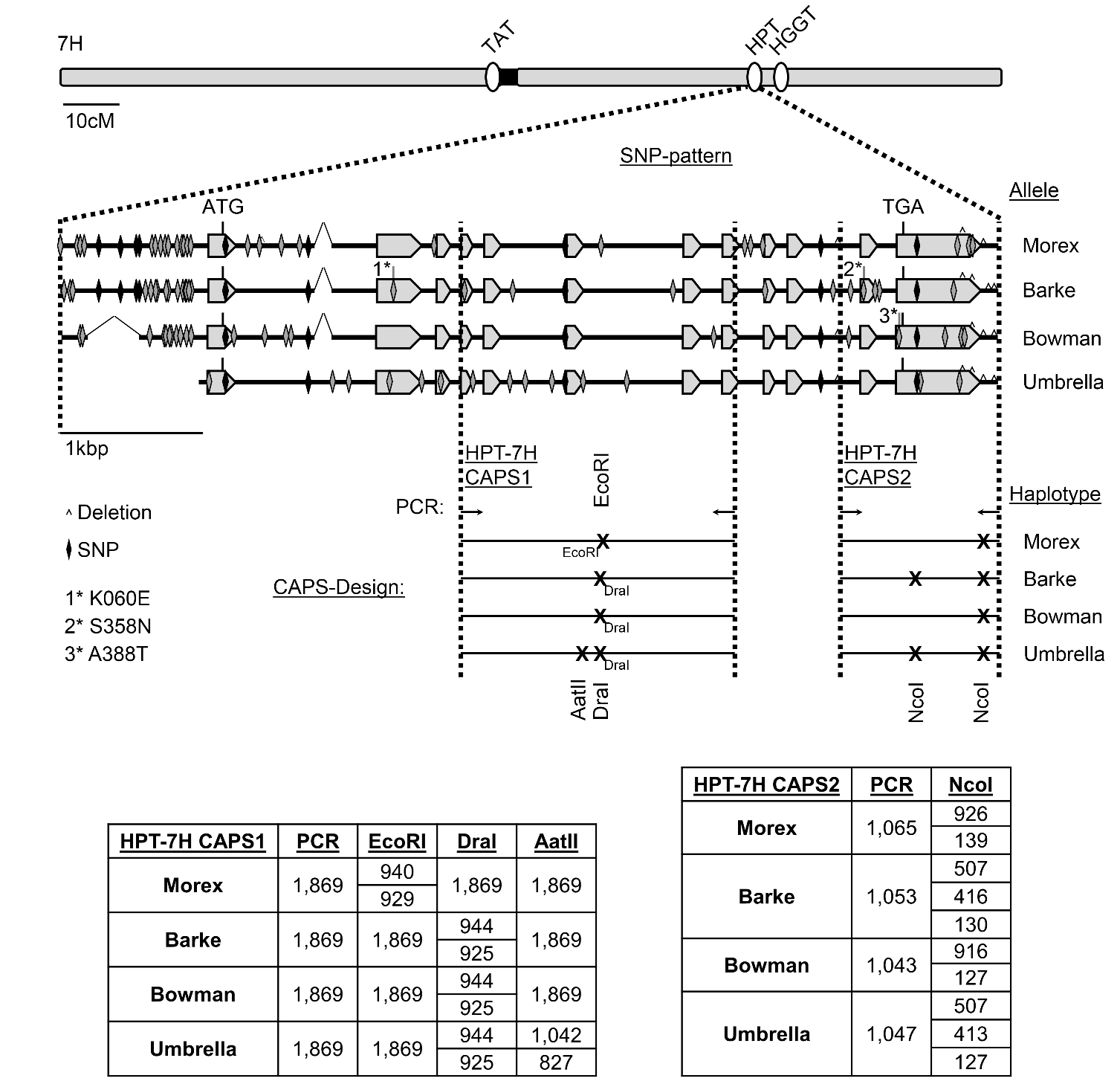


**Fig. S3: SNP distribution and CAPS marker design for the four *HPT-7H* alleles**

Chromosome arms of the barley chromosome 7H are displayed as grey bars, the centromere region as black junction. The positions of the genes are based on map position in centimorgan (cM) according to the IBSC genetic map (see text). Grey bars in the indicated *HPT-7H* locus represent exons based on cDNA information. The genomic region of the *HPT-7H* was solved by Sanger-sequencing after stepwise amplification by PCR (see also Supplementary **Fig. S2**). All four alleles were sequenced in the area as indicated. SNP positions are represented by grey rhombi (allele-specific) and black rhombi (at least two alleles share this haplotype). Three SNPs change the primary protein sequence of one allele compared to the other three (marked with asterisks). Circumflexes represent gaps bigger than one bp. Below: Two CAPS markers were designed to facilitate screening of additional genotypes. The sizes [bp] of the PCR products and restriction fragments of the individual CAPS markers are listed in the tables. Please see Supplementary **Tab. S4** online for compiled allele sequences.


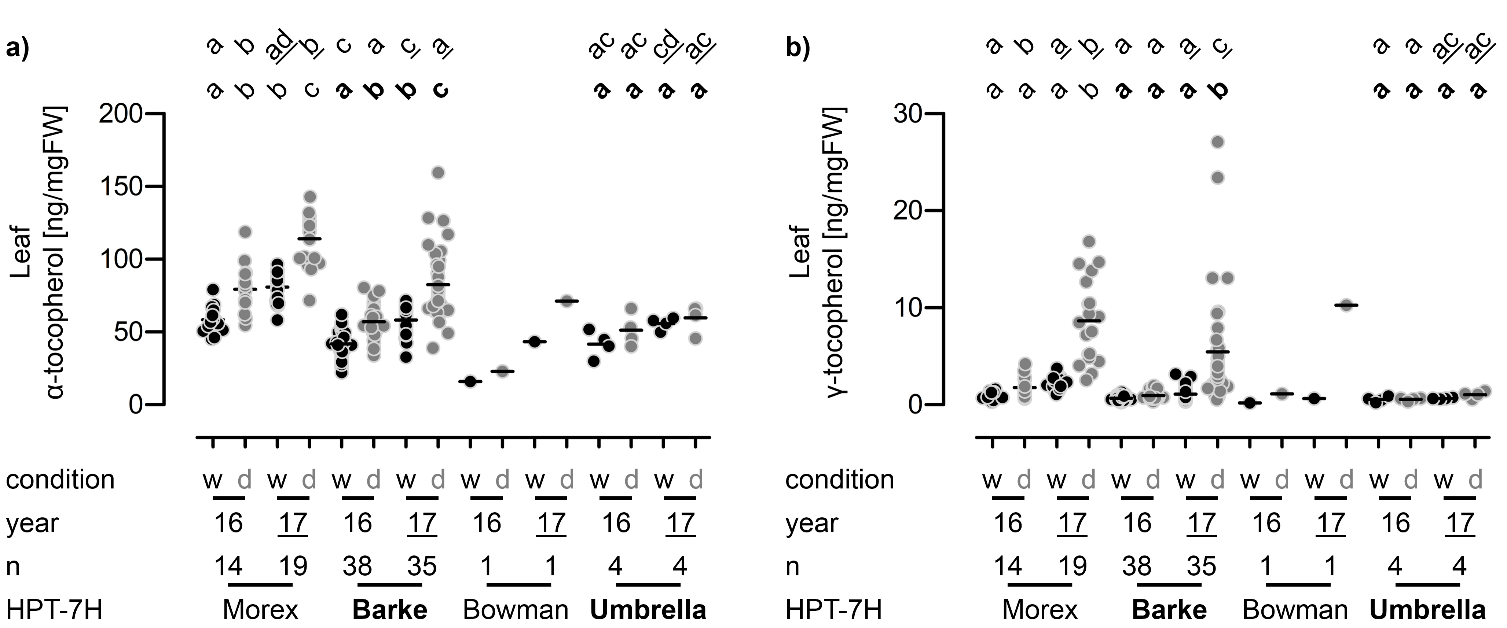


**Fig. S4: Leaf tocopherol content of barley genotypes grouped by *HPT-7H* haplotype**

The content [ng/mgFW] of **(a)** α- and **(b)** γ-tocopherol of flag leaves minus one of barley plants after heading (BBCH-stage 59-77) grown in the RS under well-watered (w – black dots) and dry conditions (d – grey dots) in 2016 (57 genotypes) and 2017 (59 genotypes) was analysed by HPLC. Each dot represents one genotype; genotypes are grouped according to *HPT-7H* haplotypes as determined by CAPS-markers (please see manuscript text and Supplementary **Fig. S3**). n: number of genotypes harbouring each haplotype. Black horizontal lines: arithmetic means. Significant differences (p < 0.05) are indicate by unequal letters and were calculated in a 1-way ANOVA followed by a pairwise Bonferroni post hoc test for all haplotypes in 2016 (top row – regular letters) and in 2017 (top row – underlined letters) as well as for each haplotype covering both years (bottom row – four characters each). For the single genotype with the Bowman haplotype, no ANOVA was possible. δ-tocopherol was only detectable in traces and is not displayed. Please see Supplementary **Tab. S3** online for raw data.


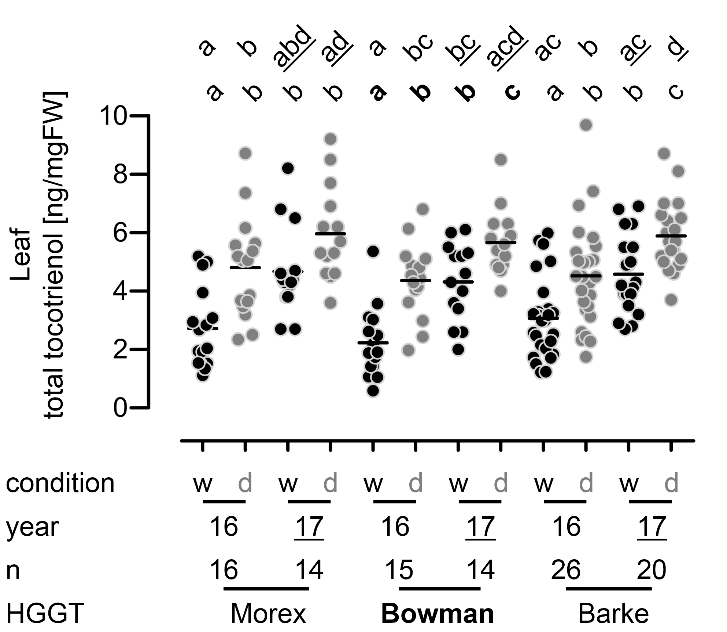


**Fig. S5: Leaf total tocotrienol content of barley genotypes grouped by *HGGT* haplotype**

The content [ng/mgFW] of total tocotrienol (sum of γ- and α-tocotrienol) of flag leaves minus one of barley plants after heading (BBCH-stage 59-77) grown in the RS under well-watered (w – black dots) and dry conditions (d – grey dots) in 2016 (57 genotypes) and 2017 (48 genotypes) was analyzed by HPLC. Each dot represents one genotype; genotypes are grouped according to *HGGT* haplotypes as determined by CAPS-markers (please see manuscript text and Supplementary **Fig. S10**). n: number of genotypes harboring each haplotype. Black horizontal lines: arithmetic means. Significant differences (p < 0.05) are indicated by unequal letters and were calculated in a 1-way ANOVA followed by a pairwise Bonferroni post hoc test for all haplotypes in 2016 (top row – regular letters) and in 2017 (top row – underlined letters) as well as for each haplotype covering both years (bottom row – four letters each). Please see Supplementary **Tab. S3** online for data for subspecies and raw data.


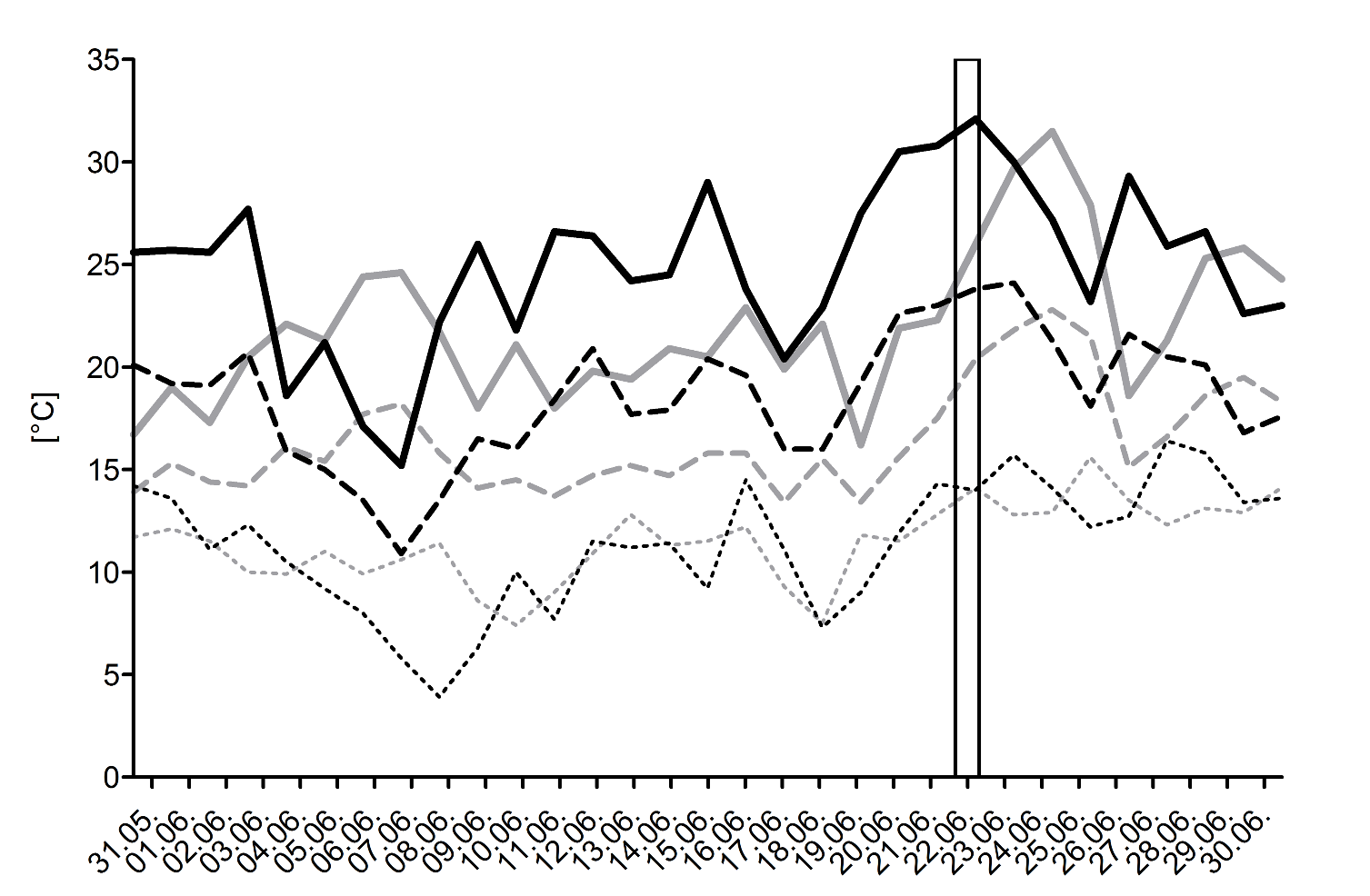


**Fig. S6: Temperature records at the field site in Freising, Germany**

The maximum (solid lines), average (dashed lines) and minimum (dotted lines) daily temperatures at the field site in Freising (Germany) were logged in 2016 (grey lines) and 2017 (black lines) at the weather station Freising No. 8 approximate 300 m away from the RS. Date of leaf sampling in both years: 22^nd^ of June (open box). In the four weeks prior leaf sampling the daily medium temperature was elevated by 2.7 °C in average in 2017 compared to 2016. Especially in the few days prior leaf sampling the maximum daily temperature was particularly increased by 6-11 °C in 2017 compared to 2016. Please see Supplementary **Tab. S5** online for raw data.


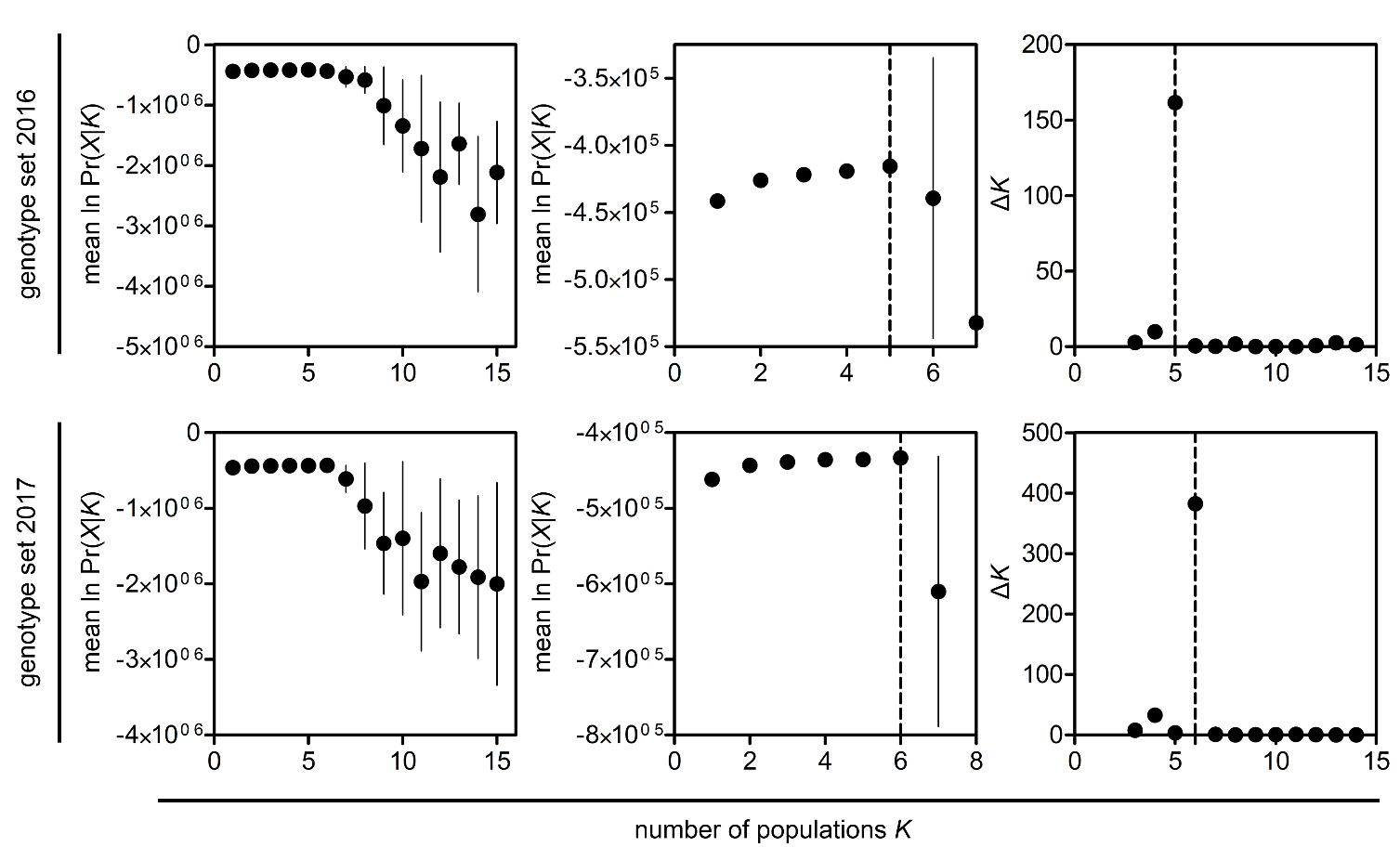


**Fig. S7: Estimation of population size**

The population structure for both sets of genotypes was inferred using a model-based approach implemented in the program STRUCTURE using 4320 informative SNPs spread throughout the genome. The mean posterior probability of *K* (ln Pr(*X*|*K*)) plus standard deviation was plotted against the number of populations (*K*) (left column) after 20 iterations. ln Pr(*X*|*K*) reaches a stable plateau at *K* = 5 (2016 – top row) and *K* = 6 (2017 – bottom row) which one recognizes better in a closeup (middle column). The highest Δ*K* value also can be found at *K* = 5 (2016) and *K* = 6 (2017). Therefore, these *K*-values were chosen for subsequent analysis. Please see Supplementary **Tab. S6** online for raw data.


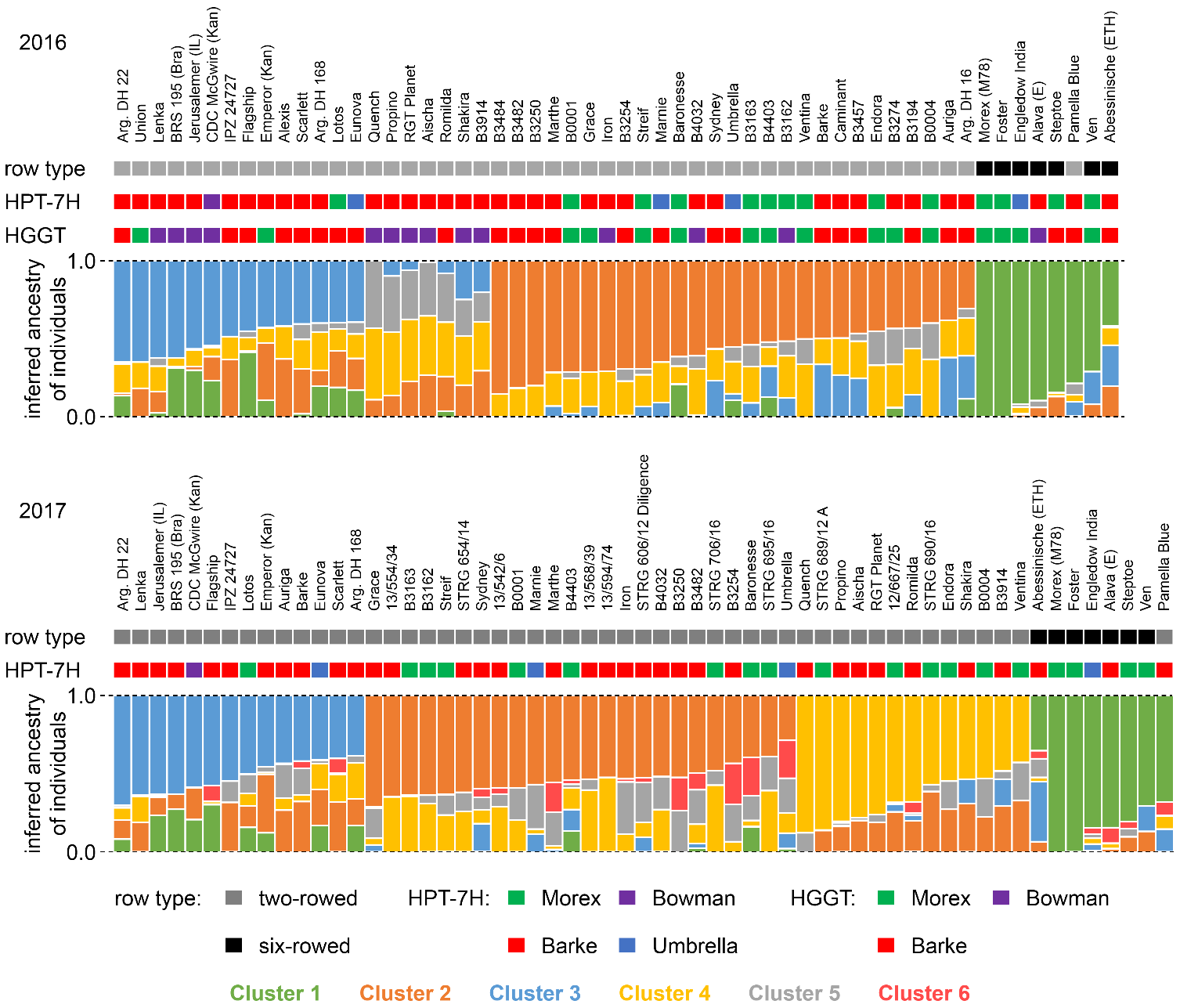


**Fig. S8: Distribution of *HPT-7H* and *HGGT* haplotype to inferred population clusters.**

The population structure for all genotypes in 2016 (*K* = 5) and 2017 (*K* = 6) was inferred using a model-based approach implemented in the program STRUCTURE using 4320 informative SNPs spread throughout the genome. The mean inferred ancestry of individuals (the estimated membership coefficients for each genotype in each cluster) is shown for all genotypes after 20 iterations. The inferred clusters are displayed in different colours. The row-type of each genotype as well as *HPT-7H* and *HGGT* haplotypes as determined by CAPS-markers (please see manuscript text and Supplementary **Fig. S3 + S10**) are also represented by a colour code given below the graph. The genotypes are sorted by the inferred ancestry as provided by STRUCTURE. Please see Supplementary **Tab. S3** online for raw data.


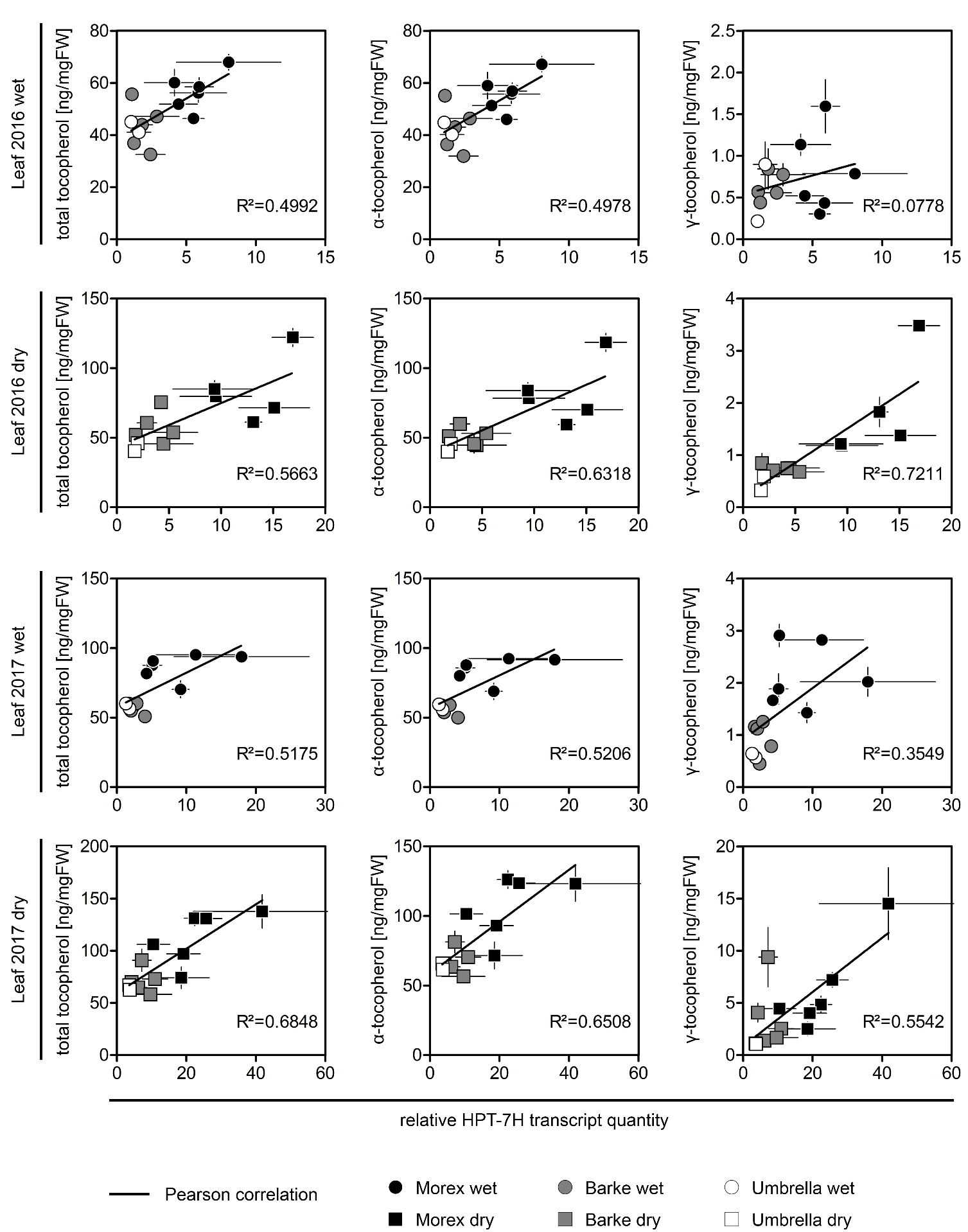


**Fig. S9: The *HPT-7H* expression level in barley leaves correlates with the tocopherol content**

The content [ng/mgFW] of total tocopherol (sum of δ-, γ- and α-tocopherol) (left column), α-tocopherol (middle column) and γ-tocopherol (right column) of flag leaves minus one of barley plants after heading (BBCH-stage 59-77) grown in the RS under well-watered (round data points) and dry conditions (square data points) in 2016 (row 1+2) and 2017 (row 3+4) was analysed by HPLC and plotted against the relative *HPT-7H* transcript quantity as determined by RT-qPCR. Each data point represents one genotype shaded according to *HPT-7H* alleles as determined by Sanger sequencing (black: Morex, grey: Barke, open: Umbrella). Bars: standard error (n = 4). The black lines indicate the Pearson correlation which was calculated independent of genotype and growth condition. Please see Supplementary **Tab. S3** online for raw data.


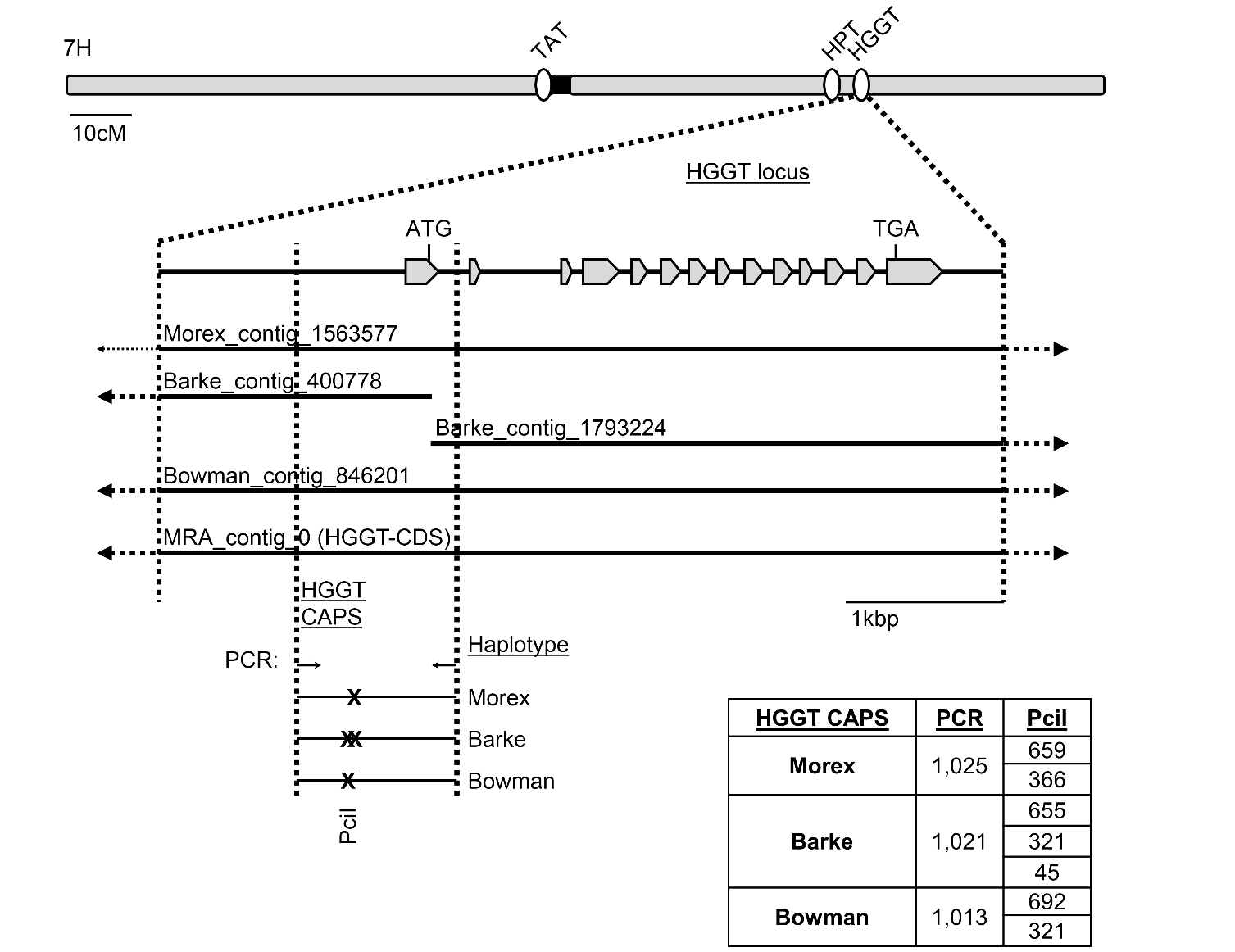


**Fig. S10: Genomic locus of the *HGGT* with CAPS design**

Chromosome arms of the barley chromosome 7H are displayed as grey bars, the centromere region as black junction. The positions of the genes are based on map positions in centimorgan (cM) according to the IBSC genetic map (see text). Grey bars in the indicated *HGGT* locus represent exons based on cDNA information. The genomic locus of the *HGGT* as it is depicted here is based on available genomic DNA data. The genomic sequence information of the WGS_contigs of the three barley cultivars Morex, Barke and Bowman as well as the pseudomolecule 7H of the MRA were used as templates. A CAPS marker was designed to facilitate screening of additional genotypes. The sizes [bp] of the PCR products and restriction fragments of the CAPS marker are listed in the tables.


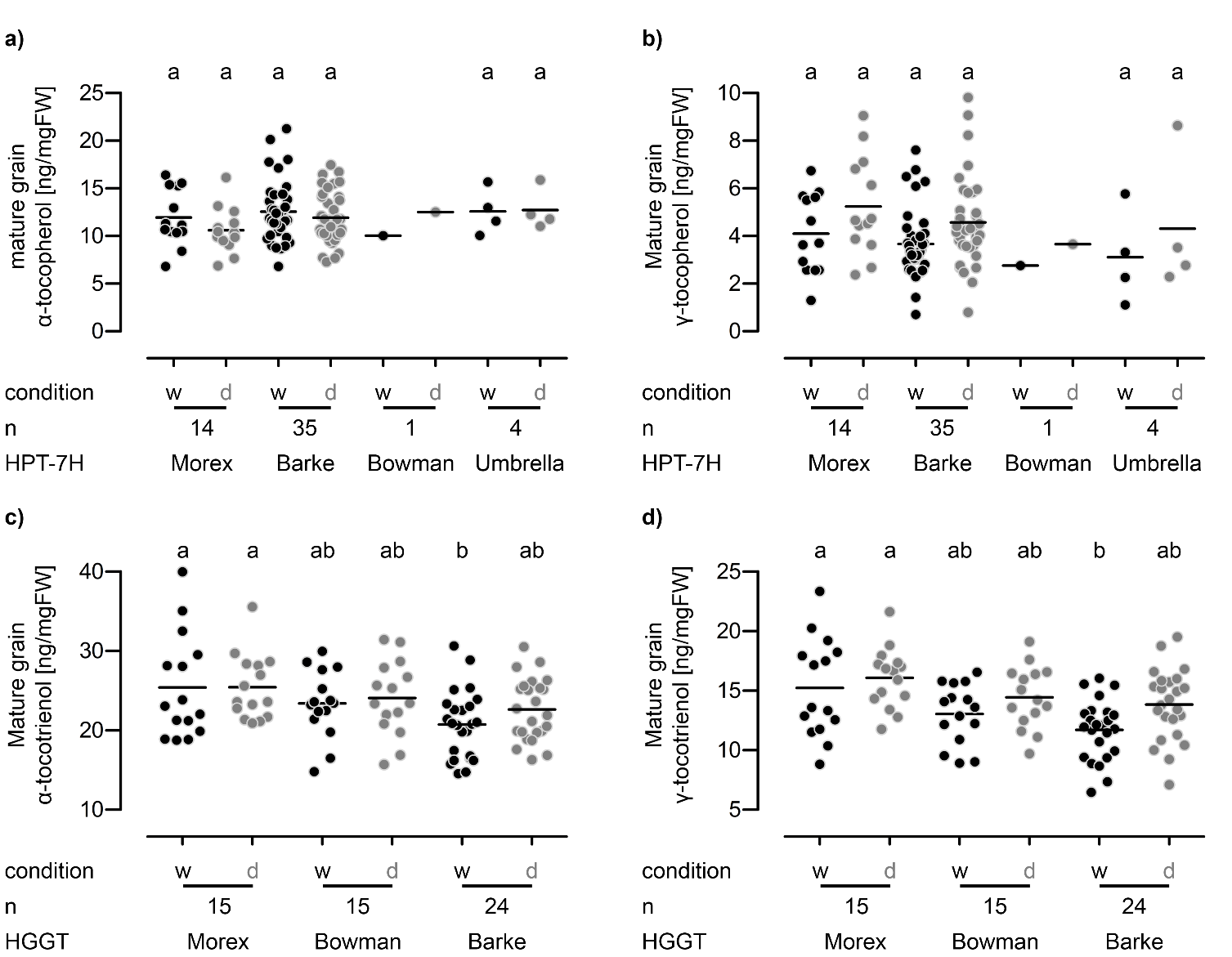


**Fig. S11: Tocopherol and tocotrienol content of mature barley grains**

The content [ng/mgFW] of **(a)** α-tocopherol **(b)** γ-tocopherol **(c)** α-tocotrienol and **(d)** γ-tocotrienol of mature barley grains of plants grown in the RS under well-watered (w – black dots) and dry conditions (d – grey dots) in 2016 was analysed by HPLC. Each dot represents one genotype; genotypes are grouped according to **(a+c)** *HPT-7H* haplotypes and **(b+d)** *HGGT* haplotypes as determined by CAPS-markers (please see manuscript text and Supplementary **Fig. S3 + S10**). n: number of genotypes harbouring each haplotype. Black horizontal lines: arithmetic means. Significant differences (p < 0.05) are indicated by unequal letters and were calculated in a 1-way ANOVA followed by a pairwise Bonferroni post hoc test for all haplotypes in each graph. For the single genotype with the Bowman *HPT-7H* haplotype, no ANOVA was possible. δ-tocopherol was only detectable in traces and is not displayed. Please see Supplementary **Tab. S3** online for raw data.


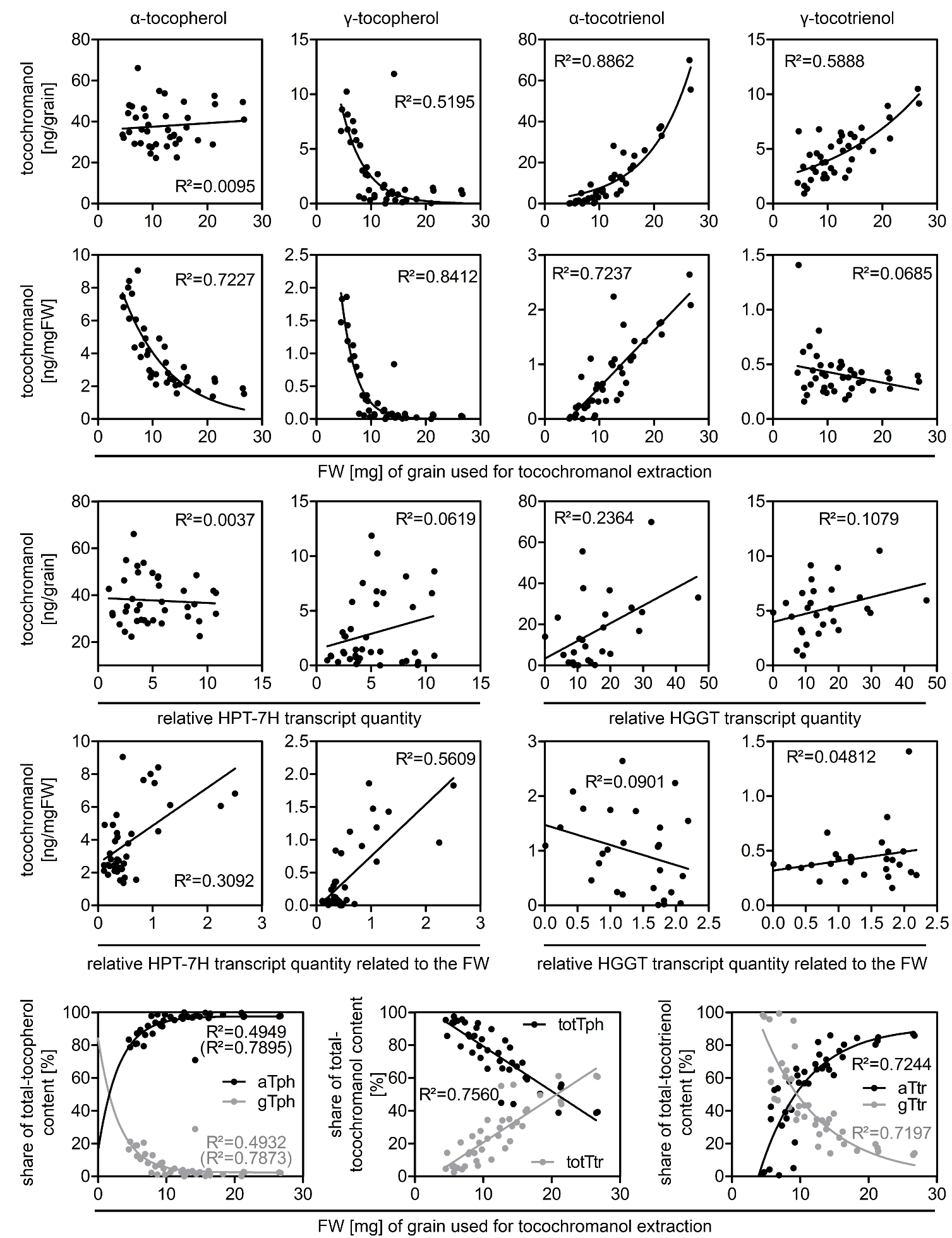


**Figure S12: Tocochromanol accumulation and relative quantities of *HPT-7H* and *HGGT* transcript levels during seed development**

We determined the relative transcript quantities of *HPT-7H* and *HGGT* and the tocochromanol content of immature still growing grains (milky to early dough stage, BBCH-stage 73-83). Each grain could either be used for RNA or tocochromanol extraction. To be able to relate transcript level to tocochromanol content we always used pairs of grains with comparable developmental stage: see **Fig. 6a+b** for further details. A total of 40 pairs of grains from 10 genotypes grown under the two conditions in the RS were analysed. Absolute amounts of tocochromanol subspecies (from left to right: α-tocopherol, γ-tocopherol, α-tocotrienol, γ-tocotrienol) extractable from each grain [ng/grain] plotted against grain age (first row). Tocochromanol concentration [ng/mgFW] plotted against grain age (second row). Absolute amounts of tocochromanol subspecies extractable from each grain [ng/grain] plotted against the relative *HPT-7H* and *HGGT* transcript quantities (third row). Tocochromanol concentration [ng/mgFW] plotted against the relative *HPT-7H* and *HGGT* transcript quantities in relation to the age (fourth row). Contribution of tocochromanol species (aTph: α-tocopherol, gTph: γ-tocopherol, totTph: total-tocopherol, totTtr: total-tocotrienol, aTtr: α-tocotrienol, gTph: γ-tocotrienol) to total tocochromanol pool (fifth row). R² in brackets: calculated without the single outlier. Please see Supplementary **Tab. S3** online for raw data.


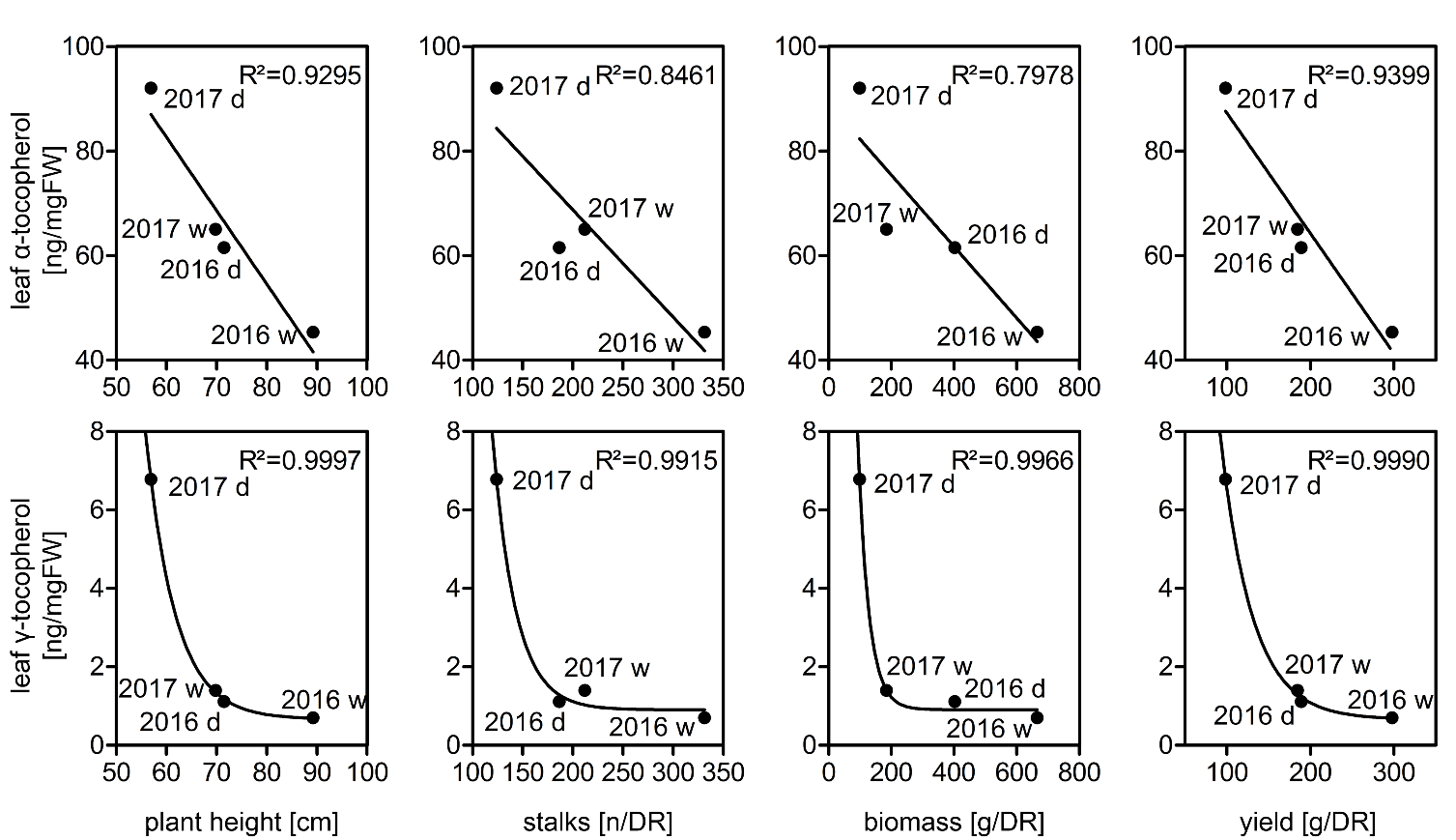


**Figure S13:** **Connection of leaf tocopherol content with morphological parameters**

Morphological parameters off barley plants grown in the RS under well-watered (w) and dry (d) conditions in 2016 (57 genotypes) and 2017 (59 genotypes) were determined as well as the contents [ng/mgFW] of α- (top row) and γ-tocopherol (bottom row) in flag leaves minus one after heading (BBCH-stage 59-77). Each data point represents the mean of all cultivars. Number of stalks [n], total biomass [g] and kernel yield [g] are depicted as values per double row (DR). The tocopherol content correlates inversely with overall growth performance and yield and might therefore be taken as an indicator for the degree of stress. The correlation appears linear for α-tocopherol and exponential for γ-tocopherol due to the strong accumulation of γ-tocopherol in 2017 under dry conditions.


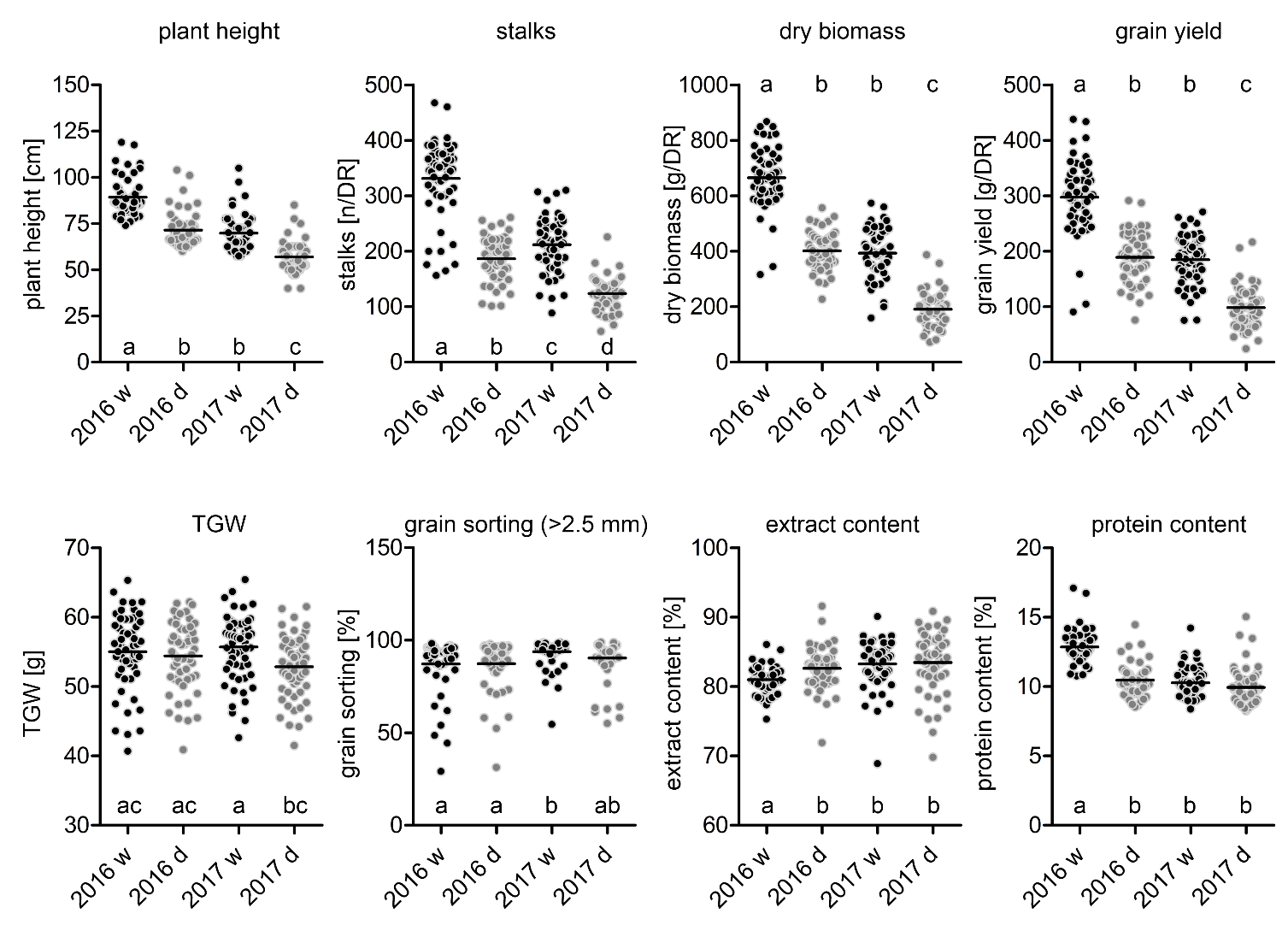


**Figure S14: Growth performance, yield quantity and quality in the 2016 and 2017 field experiments**

Growth and yield parameters for barley plants grown in the RS under well-watered (w – black dots) and dry conditions (d – grey dots) in 2016 (57 genotypes) and 2017 (59 genotypes). Each dot represents one genotype. Black horizontal lines: arithmetic means. Significant differences (p < 0.05) are indicated by unequal letters and were calculated in a 1-way ANOVA followed by a pairwise Bonferroni post hoc test. Number of stalks [n], total biomass [g] and kernel yield [g] are depicted as values per double row (DR). Plant height and tillering behavior (stalks) decreased due to increasing abiotic stress (Supplementary **Fig. S1 + S6**) resulting in decreased dry biomass and grain yield. Yield quality parameters like thousand grain weight (TGW), grain sorting, extract content and protein content were least affected by the stress conditions when comparing all genotypes.
